# Insights into targeting LKB1 in tumorigenesis

**DOI:** 10.1016/j.gendis.2024.101402

**Published:** 2024-08-28

**Authors:** Charles B. Trelford, Trevor G. Shepherd

**Affiliations:** a The Mary & John Knight Translational Ovarian Cancer Research Unit, London Regional Cancer Program, London, ON N6A 4L6, Canada; b Department of Anatomy and Cell Biology, Schulich School of Medicine and Dentistry, Western University, London, ON N6A 3K7, Canada; c Department of Oncology, Schulich School of Medicine and Dentistry, Western University, London, ON N6A 3K7, Canada; d Department of Obstetrics and Gynaecology, Schulich School of Medicine and Dentistry, Western University, London, ON N6A 3K7, Canada

**Keywords:** AMPK, LKB1, Peutz-Jeghers syndrome, STK11, Tumor suppressor

## Abstract

Genetic alterations to serine-threonine kinase 11 (*STK11*) have been implicated in Peutz-Jeghers syndrome and tumorigenesis. Further exploration of the context-specific roles of liver kinase B1 (LKB1; encoded by *STK11*) observed that it regulates AMP-activated protein kinase (AMPK) and AMPK-related kinases. Given that both migration and proliferation are enhanced with the loss of LKB1 activity combined with the prevalence of *STK11* genetic alterations in cancer biopsies, LKB1 was marked as a tumor suppressor. However, the role of LKB1 in tumorigenesis is paradoxical as LKB1 activates autophagy and reactive oxygen species scavenging while dampening anoikis, which contribute to cancer cell survival. Due to the pro-tumorigenic properties of LKB1, targeting LKB1 pathways is now relevant for cancer treatment. With the recent successes of targeting LKB1 signaling in research and clinical settings, and enhanced cytotoxicity of chemical compounds in LKB1-deficient tumors, there is now a need for LKB1 inhibitors. However, validating LKB1 inhibitors is challenging as LKB1 adaptor proteins, nucleocytoplasmic shuttling, and splice variants all manipulate LKB1 activity. Furthermore, STE-20-related kinase adaptor protein (STRAD) and mouse protein 25 dictate LKB1 cellular localization and kinase activity. For these reasons, prior to assessing the efficacy and potency of pharmacological candidates, the functional status of LKB1 needs to be defined. Therefore, to improve the understanding of LKB1 in physiology and oncology, this review highlights the role of LKB1 in tumorigenesis and addresses the therapeutic relevancy of LKB1 inhibitors.

## Introduction

Genetic screening patients diagnosed with Peutz-Jeghers syndrome (PJS) first linked serine-threonine kinase 11 (*STK11*), hereafter liver kinase B1 (*LKB1*) mutations to disease.^1^ Since linking *LKB1* mutations to PJS, *LKB1* inactivation has induced tumor formation in numerous animal studies,^2^ and sporadic *LKB1* mutations are frequently detected in solid tumors.^3^^,^^4^ Given the overwhelming evidence that genetic inactivation of *LKB1* induces tumor growth^5^, ^6^, ^7^ and overexpression decreases microvessel density,^8^ tumor burden,^9^ and cell proliferation,^10^ *LKB1* is a widely accepted tumor suppressor. Contrary to these findings, some investigations conclude that LKB1 contributes to tumorigenesis through activating reactive oxygen species (ROS) scavenging, DNA repair machinery, and autophagy.^11^, ^12^, ^13^

The paradoxical role of LKB1 in tumor biology in conjunction with the pre-clinical success of pharmacological agents targeting LKB1 pathways^14^, ^15^, ^16^ suggests a need for more precise modeling of LKB1 signaling in physiology and tumor biology. Generating LKB1-specific modulators is problematic because, in some contexts, agonists of LKB1 signaling are therapeutic,^17^ whereas other reports have linked these agonists to aggressive tumor phenotypes.^18^^,^^19^ Alternatively, due to the label of tumor suppressor, few have tried to synthesize LKB1 antagonists. Given that current strategies targeting LKB1 pathways are indirect, and few pharmacological agents become eligible for market approval,^20^ developing LKB1-specific modulators with minimal cytotoxicity is of utmost importance. Therefore, this review addresses the biochemical processes dictating LKB1 activity in cell biology and pathology and the therapeutic relevancy of targeting LKB1 activity.

## Linking *LKB1* mutations to disease

### LKB1 mutations in familial PJS

PJS is an autosomal dominant disorder characterized by hyperpigmented macules, benign gastrointestinal hamartomatous polyps, and an increased risk of malignancy.^21^, ^22^, ^23^ Hemminki et al in 1997 were the first to use comparative genomic hybridization and loss-of-heterozygosity analyses to localize a gene implicated in PJS. Comparative genomic hybridization assessed copy number variations in DNA extracted from 16 hamartomatous polyps while a loss-of-heterozygosity analysis using previously identified microsatellite markers of 19p (Dl9S886, D19S894, D19S413, and Dl9S565)^24^ as a reference revealed that PJS patients had variable chromosome 19p sequence lengths.^25^ Linkage analysis assessed the distances between the 19p microsatellite markers of 12 PJS families, which identified a susceptible locus between D19S886 and D19S883. Database searches and solution hybridization of complementary DNA (cDNA) identified 27 transcripts between D19S886 and D19S883, and *LKB1* was among them.^1^ In some PJS patients, the *LKB1* PCR products were truncated suggesting genetic deletions.^25^ Yet, for many PJS patients, the PCR products were not aberrant.^26^^,^^27^ Instead, these patients contained point mutations or frameshift mutations that produced a stop codon prematurely, disrupted intron splice acceptor sites, or shifted the reading frame.^28^^,^^29^ Only 1 of the 12 PJS families did not show *LKB1* sequence variation, which may have been due to incomplete sequencing, insensitivity of the mutation detecting methods, or large genomic deletions that were not detected by PCR.^1^^,^^30^ Alternatively, unidentified mutations may induce PJS as an analysis of 34 PJS families detected *LKB1* germline mutations in only 70% of cases.^31^ Indeed, linkage analysis linked chromosome 19q markers to a minority of PJS families,^32^ but despite the identification of a possible second locus on chromosome 19q, *LKB1* remains the only gene linked to PJS.

### LKB1 mutations in tumorigenesis

**Figure 1:**
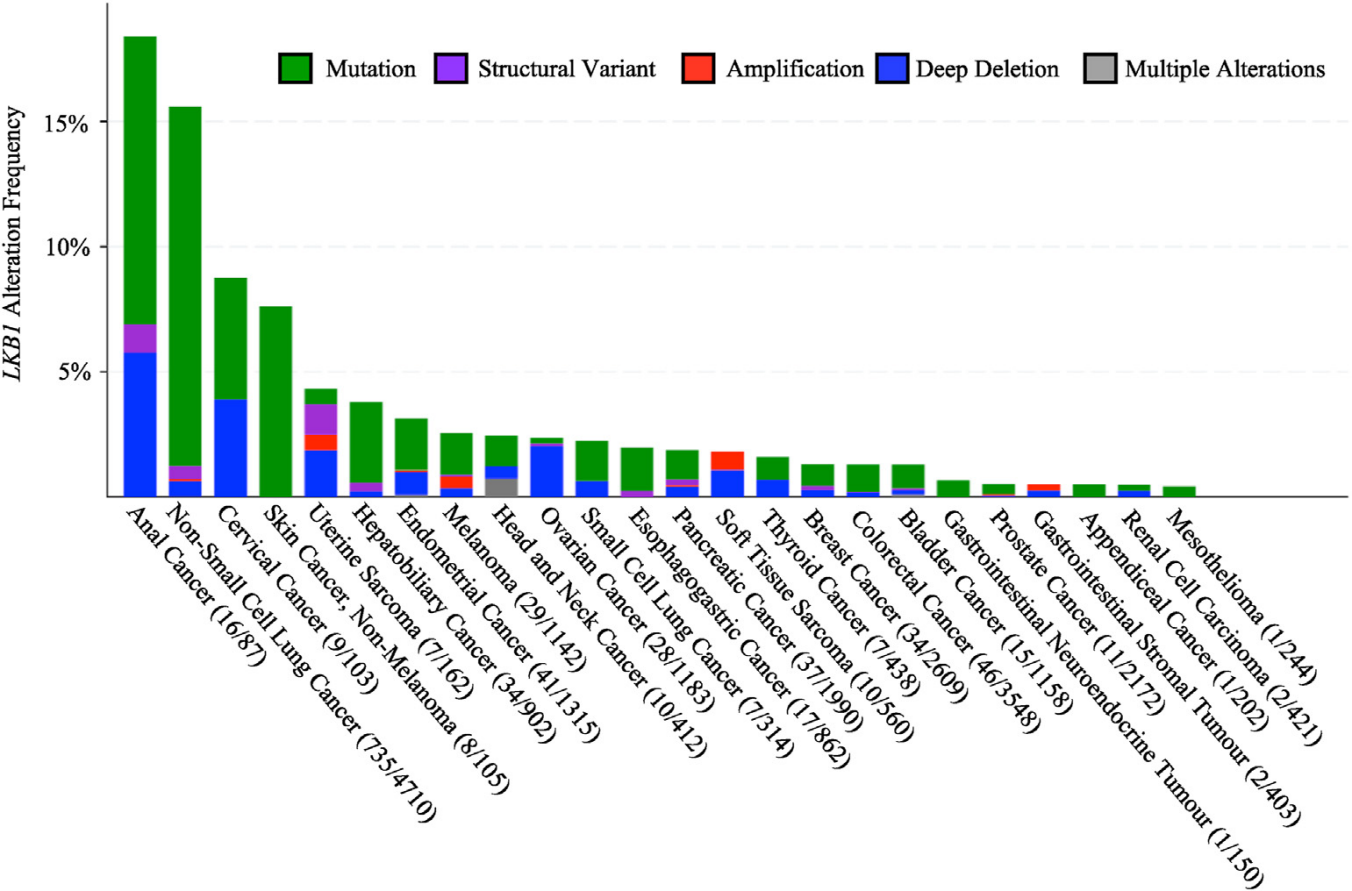
Prevalence of *LKB1* genetic alterations in human cancers. The alteration frequency including mutations, structural variants, amplifications, deep deletions, and multiple alterations of *LKB1* in tumors. Listed in descending prevalence are *LKB1* genetic alterations with the number of genetic alterations and total specimens tested in brackets. The data was obtained using cBioPortal for Cancer Genomics of the pan cancer study entitled MSK MetTropism (MSK, *Cell* 2021^41^).

Although heterozygous germline mutations in *LKB1* are implicated in PJS, the two-hit hypothesis suggests that a second allele must be genetically altered to induce tumor formation,^33^ which explains why PJS increases cancer frequency but does not guarantee malignancy.^34^ Subsequent investigations revealed that LKB1 was the first kinase in which inactivating its kinase activity increased the risk of tumorigenesis.^27^ Indeed, sequencing biopsies of intestinal polyps and pancreatic cancer confirmed that PJS patients are more susceptible to tumor formation as only the cancerous tissues contained additional somatic mutations to the remaining *LKB1* allele.^35^ In addition to pancreatic cancer, *LKB1* somatic mutations, structural variants, amplifications, and deletions have been described in many cancer types including lung adenocarcinoma,^36^ hepatocellular carcinoma,^37^ head and neck squamous cell carcinoma^38^ as well as cancers of the colon,^39^ cervix,^4^ and breast^40^ (Fig. 1).^41^

Despite the incidence of *LKB1* mutations being relatively uncommon in most cancers,^42^ genomic analyses routinely detect *LKB1* mutants in approximately 10%–30% of non-small cell lung cancer patients.^43^ In fact, the only mutations in lung adenocarcinoma with a greater incidence than *LKB1* are mutations to Kristen rat sarcoma viral oncogene homolog (*KRAS*) and tumor protein p53 (*TP53*).^44^ In many cases, genomic investigations found that *LKB1* mutations coincided and synergized with other mutations.^45^ For instance, approximately half of *KRAS*^*G12D*^ driven lung cancers inactivate *LKB1*.^46^ In addition to *KRAS*, *LKB1* is often co-mutated with *TP53*^47^ and *KEAP1*.^48^ From a clinical standpoint, understanding the synergy of *LKB1* co-mutants may predict patient prognosis as the overall survival of patients with *LKB1/KRAS* co-mutant tumors is poor compared with other *LKB1* co-mutant tumors.^47^ In fact, *LKB1* mutations are prognostic biomarkers that may influence treatment regimens as immune checkpoint blockade inhibitors are less effective in *LKB1/KRAS* mutant tumors.^48^ A potential explanation is that *LKB1* inactivation up-regulates DNA methyltransferases to silence stimulator of interferon genes, which is associated with poor immune responses and aggressive tumor phenotypes.^49^

### AMPK and AMPK-related kinases (ARKs) dictate the role of LKB1 in tumorigenesis

Searching for LKB1 substrates responsible for its tumor suppressing functions revealed that 5′ adenosine monophosphate-activated protein kinase (AMPK) and AMPK-related kinases (ARKs) are phosphorylated by LKB1.^50^^,^^51^ Indeed, most mammalian tissues rely on LKB1 to activate AMPK during oxidative and metabolic stress^52^ to regulate a broad scope of cellular processes including cell division,^53^ metabolism,^54^ growth,^55^ polarity,^56^ energy expenditure,^57^ transcription,^58^ DNA repair,^59^ and apoptosis.^60^ As such, in many patient specimens and animal models, the loss of LKB1-AMPK signaling alters cell growth and repair mechanisms triggering cancer development.^61^, ^62^, ^63^ Furthermore, AMPK agonists and pharmacological compounds that activate AMPK, such as cafestol and β-ionone disrupt tumor progression.^64^^,^^65^ Alternatively, LKB1-AMPK signaling enhances tumor cell survival through NADPH-mediated ROS scavenging,^66^ blocking tumor cell anoikis during basement membrane detachment,^67^ and activating autophagy to sustain metabolism in tumor cells to mediate proliferation.^68^

In addition to AMPK, ARKs such as microtubule affinity-regulated kinases (MARKs), salt inducible kinases (SIKs), NUAK family kinases (NUAKs), and sucrose non-fermenting-related kinase (SNRK) have been linked to tumor suppressing or promoting functions of LKB1. For instance, gene expression signatures were similar between *LKB1*- and *SIK*-deficient lung tumors indicating that these proteins both regulate tumorigenesis through the same pathways.^69^ In fact, KRAS-dependent non-small cell lung cancer mouse models revealed that the conditional knockout of *SIK1* and *SIK3* accelerated tumor growth.^70^ LKB1-SIK signaling also maintains the activity of the oncogenic transcription factor MEF2C (myocyte-specific enhancer factor 2C) in acute myeloid leukemia linking LKB1-SIK activity to cancer.^71^ Furthermore, LKB1-NUAK1 activity augments tumor invasion by promoting cell detachment and activating cytoskeletal motor proteins.^72^ Elevated *NUAK1* expression is also associated with poor pancreatic ductal adenocarcinoma survival as inactivating NUAK1 reduces pancreatic cancer proliferation.^73^ Alternatively, LKB1-MARK signaling suppresses high-grade ovarian cancer development by reducing angiogenesis and cell growth.^74^ Finally, SNRK decreases β-catenin protein levels and activity in colon cancer, which decreases tumor cell proliferation.^75^ Therefore, there is a duality to both LKB1-AMPK and LKB1-ARK signaling in tumorigenesis as different downstream pathways either augment or antagonize tumor progression.

### Animal models linking LKB1 to disease

*In vivo* investigations that analyze LKB1-AMPK activity on cell polarity and migration rely on administering tamoxifen to tissue-specific Cre-Lox transgenic mouse models because germline knockouts of *LKB1*, *AMPK*, or their respective homologs are lethal in *Caenorhabditis elegans*^76^, *Drosophila melanogaster*,^77^ and murine models.^55^ Tissue-specific Cre-Lox *LKB1* murine knockout models have identified many pathologies driven by mutational inactivation of *LKB1* including cardiac defects,^78^ metabolic disorders,^79^ cachexia,^80^ kidney disease,^81^ hormonal imbalances,^82^ and tumorigenesis^2^^,^^83^, ^84^, ^85^ (Table 1).

**Table 1:** Non-tumorigenic and tumorigenic Cre-Lox *LKB1* knockout mouse models.

	Experimental mouse model	*LKB1* knockout tissue	Phenotype	Reference
Non-tumorigenic	Kidney-specific-CadherinCre:LKB1^flox/flox^	Kidney	Decreased expression of regulators of metabolism, kidney disease, dedifferentiated tubule epithelial cells, and dilated tubules	86
	Rosa26-Cre^ER^:LKB1^flox/flox^	All tissues	Decreased expression of regulators of metabolism, embryonic lethality, reduced body weight, hyperglycemia, glucose intolerant	57
	Myosin heavy chain ⍺Cre:LKB1^flox/flox^	Heart	Increased expression of collagen I/III, spontaneous atrial fibrillation, atrial remodelling, left ventricular hypertrophy, fibrosis, and death within 6 months of birth	82
	Muscle creatine kinaseCre:LKB1^flox/flox^	Skeletal and cardiac muscle	Myopathy, atrial dilation, decreased body weight, decreased fast twitch skeletal muscle, skeletal muscle atrophy, and loss of hindlimb function	85
	Leucine-rich repeat-containing G-protein coupled receptor 5GFP-Cre/+:LKB1^flox/flox^:R26^Td/+^	Intestinal stem cells	Restricted intestinal stem cells differentiation to secretory lineages	90
	Pro-opiomelanocortin-Cre:LKB1^flox/flox^	Pituitary gland	Altered expression of metabolic genes in the liver, diabetes, insulin resistance, and glucose intolerance.	84
	Pancreatic and duodenal homeobox 1-Cre^ERtam^:LKB1^flox/flox^	Pancreas	Glucose tolerance and resistance to hyperglycemia	91
	Transthyretin-CRE^ERT2^:LKB1^flox/flox^	Liver	Increased liver regeneration and hepatocyte proliferation	55
	Tyrosine kinase with immunoglobulin-like and epidermal growth factor-like domains 1-CRE:LKB1^flox/flox^	Endothelium	Embryonic lethal and vascular defects	92
	Empty spiracles homeobox1-Cre:LKB1^flox/flox^	Cortical neurons	Disrupted axon initiation, axon specification, neuronal proliferation, and neural development	58
	MX dynamin-like GTPase 1-Cre:LKB1^flox/flox^	Haematopoietic stem cells	Depletion of Haematopoietic stem cells	93
Tumorigenic	Keratin 14-Cre:LKB1^flox/flox^	Epidermis	Spontaneous squamous cell carcinoma	94
	Small proline rich protein 2F-Cre:LKB1^flox/flox^	Endometrial	Death and diffuse malignant endometrial cancers sensitive to mTOR inhibitors	89
	Cluster of differentiation 19-Cre:LKB1^flox/flox^	B-lymphocytes	Death and B-cell lymphoma	95
	AhER-Cre:LKB1^flox/flox^:PTEN ^flox/flox^	Epithelium and Urothelium	Bladder cancer and signs of hypoxia, EMT, and increased proliferation in the urothelium	96
	W-Myc:W-Cre:LKB1^flox/flox^	Epithelium and Urothelium	Loss of mammary gland epithelial integrity; LKB1 deletion in combination with oncogenic C-Myc induced mammary tumorigenesis	97
	Ah-Cre:LKB1^flox/flox^	Prostate	Prostate hyperplasia, prostate intraepithelial neoplasia, bulbourethral gland cysts, and hyperplasia of the urethra	98
	Pancreatic and duodenal homeobox 1-Cre:Kras^G12D^:LKB1^flox/flox^	Pancreas	Pancreatic ductal adenocarcinoma	99
	Kras^LSL−G12D/+:^ LKB1^flox/flox^	Lung	Adenocarcinoma to squamous cell carcinoma transdifferentiation. Resistant to KRAS^G12C^ inhibitors	100
	LKB1^flox/flox^/PTEN ^flox/flox^	Lung	Squamous cell carcinoma	101

Non-tumorigenic *LKB1* knockout models have historically been utilized to assess the role of LKB1 on organ/tissue development. For instance, deleting *LKB1* in somatic testicular cells produced irregular seminiferous tubules lined with only Sertoli cells, which lacked polarity, junctional complexes, and focal adhesion kinase activity.^86^ In the developing brain, LKB1 regulates neural cell migration and the development of the cerebral cortex. *LKB1* inactivation in green fluorescent protein-labeled neurons within embryonic mice increased migration to the intermediate zone but decreased migration to the middle and upper cortical plate of the cerebral cortex.^87^ Generating hindbrain *LKB1* knockout mice by crossing floxed *LKB1* mice with *Atoh1*-cre mice revealed that *LKB1* deletion decreased migration of cerebellar granule cells leading to structural abnormalities of the cerebellum.^88^ Mechanistically, LKB1 activates glycogen synthase kinase 3β at the leading edge of migrating neurons, and as a result, adenomatous polyposis coli stabilizes microtubules at the leading edge, which promotes centrosome forward movement.^89^ *AMPKB1* null mice developed using *AMPKB1* gene trap embryonic stem cells have demonstrated that AMPK is essential for mammalian brain development.^90^ However, another group utilized *AMPKA*-null cortical neurons and found that neural polarization may not require AMPK.^91^ Regardless, due to the overwhelming evidence suggesting the contrary, LKB1-AMPK signaling is essential to neuronal cell polarity, migration, and embryo development.

Given the function of LKB1-AMPK as an energy sensor, *LKB1* knockout mouse models have linked LKB1 inactivation to metabolic diseases. Indeed, female mice with POMC neuron-specific *LKB1* deletion developed phenotypes resembling type 2 diabetes including insulin resistance and glucose intolerance.^92^ Mass spectrometry analysis of newly synthesized proteins in mice with *LKB1*^*−/−*^ liver revealed that phenotypes resembling type 2 diabetes correlate with increased expression of proteins implicated in fatty acid synthesis and fatty liver.^93^ Likewise, mice with liver-specific *LKB1* deletion developed hyperglycemia post Cre injection and PCR revealed that *LKB1* loss increased the expression of genes that regulate gluconeogenesis.^94^ LKB1-AMPK signaling is also important for hormonal stimulation of glucose uptake as *LKB1* knockdown can suppress testosterone-dependent AMPK activation and glucose transport in adipocytes.^95^ In addition to pathology, muscular dysfunction is observed in the absence of LKB1 activity. Skeletal muscle-specific *LKB1*^*−/−*^ mice demonstrated reduced AMPK activation and glucose transport as well as elevated AMP:ATP ratios following contraction.^96^

LKB1-AMPK activity down-regulates genes important for lipogenesis and cholesterol synthesis including fatty acid synthase, acetyl-CoA carboxylase, and sterol regulatory element–binding protein 1.^94^ Indeed, up-regulating AMPK activity decreased atherosclerosis and hepatic steatosis in diet-induced insulin-resistant mice by antagonizing sterol regulatory element–binding proteins.^97^ Given that lipogenic enzymes induce ferroptosis, an iron-dependent non-apoptotic cell death, and lipid hydroperoxide accumulation, LKB1-AMPK protects cells from metabolic damage.^98^ The LKB1-AMPK pathway is essential to survival as viability was significantly reduced in *LKB1*-deficient zebrafish larvae during nutrient deprivation due to autophagy ablation.^99^

There have been numerous animal models linking *LKB1* inactivation to spontaneous tumor formation. For example, mice with pancreatic-specific *LKB1* inactivation developed serous cystadenomas with evidence that their pancreatic acinar cells had impaired integrity, loss of basal nuclear positioning, and absence of cell–cell junctions.^100^ Impairing the LKB1-AMPK-mTORC1 (mechanistic target of rapamycin complex 1) pathway through the conditional knockout of *LKB1* in an osteogenic mouse model induced tumor formation and bone formation and increased osteoblast differentiation and cell invasion into medullary cavities of bones.^101^ In fact, mouse models have linked LKB1 inactivation to cancers of the liver,^102^ bladder,^103^ prostate,^104^ *etc*. In support of these findings, activating the LKB1-AMPK axis attenuates epithelial–mesenchymal transition and/or metastasis in renal cell carcinoma,^105^ hepatocellular carcinoma,^106^ and cancer of the colon,^107^ breast,^108^^,^^109^ and lung.^110^

As previously discussed, LKB1/KRAS mutant lung cancer mouse models demonstrated that LKB1/KRAS-deficient tumors are more likely to develop metastases compared with other KRAS-linked mutations. Co-mutations to LKB1 and KRAS often result in mixed tumor histology linking LKB1 to cancer plasticity such as adeno-to-squamous cell-to-large cell carcinoma transdifferentiation.^111^ In LKB1/KRAS mice, the adeno-to-squamous cell carcinoma transdifferentiation is mediated by the loss of lysyl oxidase, which up-regulates p63, an oncogene linked to squamous cell survival.^112^ The clinical significance of LKB1 loss in cancer plasticity is that KRAS^G12D^ and *STK11* co-mutations have a squamous cell carcinoma gene signature linked to resistance against KRAS inhibitors.^113^ The role of LKB1 in lung cancer transdifferentiation is not limited to KRAS as LKB1 and PTEN co-mutations in adenocarcinoma acquire squamous cell carcinoma properties.^114^ As such, further investigations into the role of LKB1 in cancer plasticity will expand the current understanding of LKB1 in tumorigenesis.

Although many animal models provide evidence for the tumor suppressing functions of LKB1, there is now evidence for the contrary. Using ovarian cancer models, LKB1-AMPK activation increased tumor metastasis whereas siRNA knockdown of *AMPK* impaired peritoneal dissemination and metastasis.^115^ In fact, female NOD/SCID mice injected with *LKB1* knockout epithelial ovarian cancer cells had reduced tumor burden and metastasis through an AMPK-independent mechanism.^116^ Given these conflicting reports, more work is needed to characterize the role of LKB1 in the presence or absence of AMPK signaling. This work should aim to uncover why LKB1 may increase ovarian carcinoma invasion while dampening the invasion of other cancers.

## Linking LKB1 expression to disease

### LKB1 genetic structure

**Figure 2:**
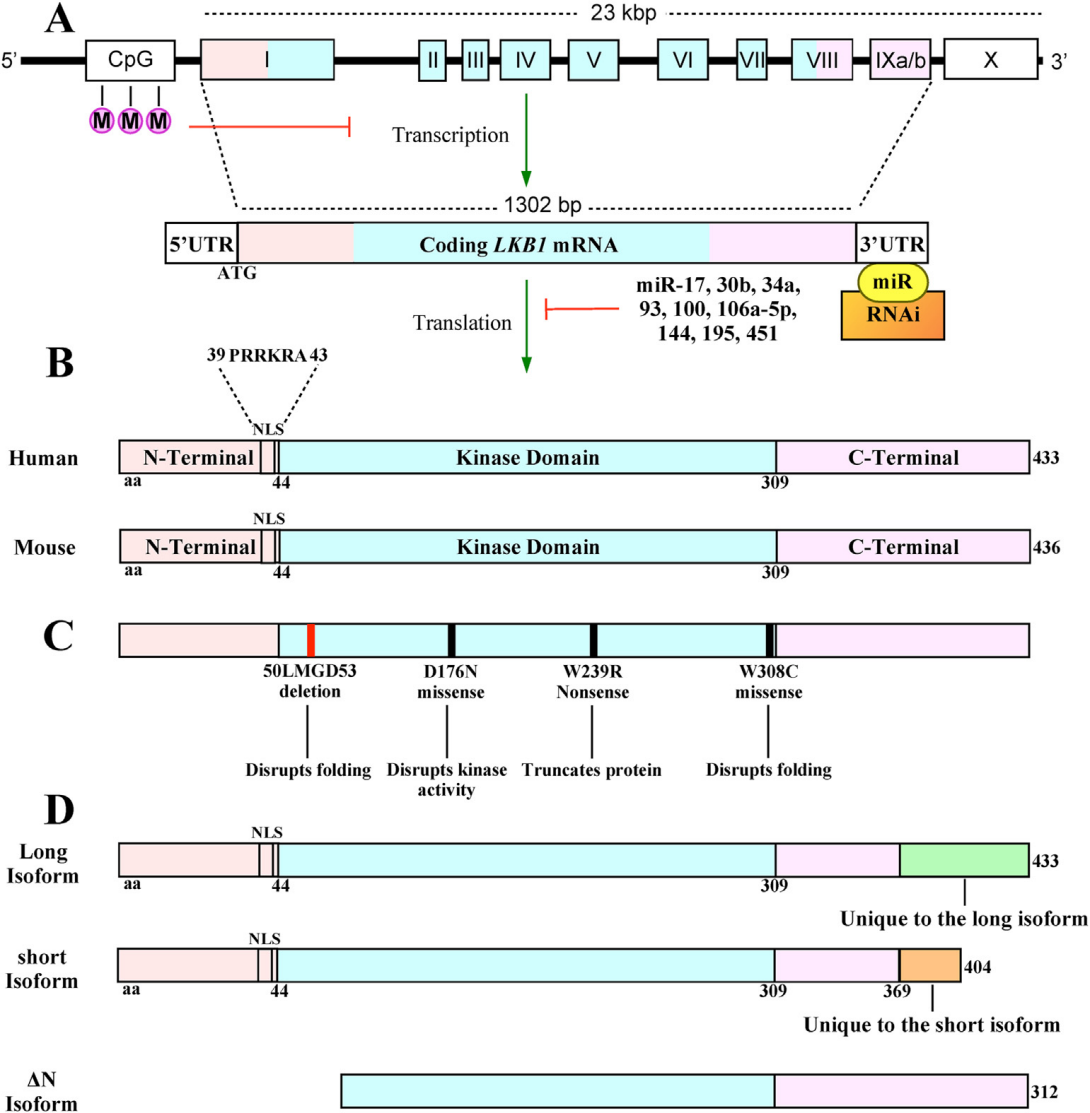
The regulation of *LKB1* DNA and mRNA. **(A)** The *LKB1* gene is 23 kbp containing 10 exons (I–X). Only exons I–IX are translated, exon I contains an ATG start codon, and exon IX has two termination signals making two alternative exon variants denoted as IXa and IXb. The *LKB1* mRNA is 1302 bp in length flanked by a 5′ and 3′ untranslated region (UTR) produced by part of exon I and the entirety of exon X, respectively. The methylation of CpG islands near the *LKB1* promoter suppresses *LKB1* transcription whereas miR-17, 30b, 34a, 93, 100, 106a-5p, 144, 195, or 451 binding to the 3′ UTR recruits RNA-induced silencing complex (RNAi) to cleave *LKB1* mRNA prior to translation. **(B)** Human and mouse LKB1 proteins are 433 and 436 amino acids (aa) long, respectively. The N-terminal domain (aa 1–43; beige) contains a nuclear localization signal (NLS) consisting of PRRKRA residues located between aa 39–43. The kinase domain (aa 44–309; aqua) is between the N-terminus and C-terminus (aa 309–; pink). The beige, aqua, and pink in the gene and mRNA correspond to the protein region. **(C)** Mutations identified in Peutz-Jeghers syndrome patients that disrupt LKB1 activity. 50 aa-LMGD-53 aa deletions disrupt protein folding, D176N missense mutation disrupts kinase activity, W239R nonsense mutation truncates the protein, and W308C missense mutation disrupts folding. Adapted from Mehenni et al, 1998.^135^**(D)** LKB1 is spliced to produce a long 433 amino acid (aa) or short 404 aa isoform. Although both long and short LKB1 isoform have an equivalent N-terminus domain, NLS, and kinase domain, they both have unique C-terminal regions (green and orange). The ΔN isoform (312 aa) is missing the N terminus and part of the kinase domain.

Jenne et al in 1998 sequenced the PJS gene susceptibility region using D19S886 microsatellite markers and chromosome 19-specific cosmid libraries in Genbank as a reference.^117^^,^^118^ Restriction digests and southern-blot analysis first mapped the D19S886 locus, which was followed by several rounds of cosmid walking (primer generation, PCR, and sequencing) revealing that human *LKB1* was approximately 190 kbp from D19S886. The exon-intron structure of *LKB1* was subsequently sequenced using the reverse transcribed 1302 bp cDNA sequence of *LKB1*^119^ as a template and primer for both PCR and sequencing. *LKB1* is comprised of 10 exons, spanning approximately 23 kbp on chromosome 19p13.3. However, only nine coding exons (exons 1–9) are transcribed in a telomere to centromere direction to produce mature LKB1 (Fig. 2A).^28^ Both human and mouse LKB1 contain an N-terminal PRRKRA (aa 38–43) motif similar to a single basic type nuclear localization signal^120^ and mutating this sequence resulted in the cytoplasmic accumulation of LKB1.^121^ Other than the serine-threonine kinase motif (aa 44–309) that is poorly related to other protein kinases, there are no other functional domains on flanking N-terminal or C-terminal regions^29^ (Fig. 2B).

### LKB1 methylation promotes tumorigenesis

Hypermethylation of the *LKB1* promoter (Fig. 2A) is linked to tumorigenesis,^122^ environmental health hazards, such as smoking,^123^ advanced stages of the tumor-node-metastasis staging, and shorter patient survival times according to Kaplan–Meier survival analyses.^124^ Methylation-specific PCR has detected *LKB1* methylation in PJS^125^ as well as renal cell carcinoma,^124^ melanoma,^126^ and colon cancer.^122^ Interestingly, LKB1 controls the expression of numerous genes through methylation as assessing an Illumina 450K microarray of lung adenocarcinoma uploaded to The Cancer Genome Atlas (TCGA) found that LKB1 loss-of-function decreased β-values representing a global reduction of CpG methylation. Indeed, methylation of 33.7% of CpG sites was decreased in *LKB1*-deficient tumors. In fact, *DNMT1* (DNA methyltransferase 1) expression is reduced by *LKB1* loss.^127^

### MicroRNAs targeting LKB1 are linked to tumorigenesis

The TCGA has been utilized to identify microRNAs (miRs) including miR-17,^128^ −30b,^129^ −34a,^130^ −93,^129^ −100,^131^ −106a-5p,^132^ −144,^133^ −195,^134^ and −451^135^ (Fig. 2A) that antagonize *LKB1* expression. For instance, there is an inverse relationship between *LKB1* and *miR-100* expression in head and neck cancer samples^131^ and *LKB1* and *miR-106a-5p* in lung adenocarcinoma.^132^ miR targeting software (TargetScan, miRBD, miRTArbase, and miRWalk) have assessed sequence alignment between *miR-17* and *LKB1* and a retrospective analysis confirmed an inverse relationship between *miR-17* and *LKB1* expression, which was verified using TCGA analyses.^128^ Nanostring nCounter technology generated a miR prediction model by quantifying miR counts using digital readouts of miR fluorescent probes that hybridize to *LKB1*. These techniques discovered that both miR-93 and miR-30b down-regulate *LKB1*.^129^ Given the evidence that increasing the expression of *LKB1*-targeting miRs contributes to tumorigenesis by down-regulating *LKB1*, targeting miRs may be a therapeutic option for tumors with low LKB1 activity that retain wild-type *LKB1* alleles. However, there is evidence that miR-17∼92 targeting of LKB1 increases tumor cytotoxicity of biguanide treatments suggesting that miR targeting of LKB1 may be beneficial in cancers reliant on LKB1 activity.^136^

### LKB1 mutations linked to PJS and tumorigenesis

While genomic analyses discovered the genetic structure of *LKB1* and contributions of *LKB1* mutations to pathology, computational algorithms in combination with constitutively updated genomic/proteomic databanks have further realized the importance of LKB1 in disease.^137^ Computational assessments of *LKB1* have accurately predicted disease loci and detected low-frequency mutations absent in sequenced patient cohorts,^138^ and in conjunction with online databases, are routinely utilized as a reference to approximate *LKB1* gene/protein structure,^139^ regulatory elements,^140^ and protein function.^141^

Initial applications of sequenced *LKB1* discerned specific genomic alterations that resulted in PJS phenotypes.^142^ Mutant *LKB1* contained deletions within introns 3, 5, and 7, deletions within coding exons 4 and 5, and inversion of exons 6 and 7. Compared with wild-type alleles, genomic deletions and rearrangements of *LKB1* sequences identified in PJS patients produced truncated polypeptides, which were later revealed to lack significant proportions of the kinase domain.^28^ More specifically, LKB1 enzymatic activity is disrupted in D176N mutants suggesting D176 is in the catalytic core where its negative charge may function to stabilize nearby residues and the phosphate group during transfer.^143^ LKB1 folding is most likely disrupted in LMGD (residues 50–53) deletion mutants because protein folding may generally accommodate deletions of surface residues^144^ or whole domains^145^ whereas LMGD deletions occur at the beginning of an internalized β-sheet. Aberrant LKB1 folding is observed in PJS patients containing the W308C missense mutation likely through mutant C308 forming a disulfide bridge with the proximal C158.^143^ Finally, W239R and W308C destabilized LKB1, decreased ATP binding capacity, and disrupted kinase activity^137^ by producing truncated proteins^138^ (Fig. 2C).

### LKB1 splicing

An RNase protection analysis has demonstrated that all mouse tissues express *LKB1* mRNA^146^ whereas northern blots of human mRNA probed with an [ɑ-^32^P] dCTP-labelled *LKB1* cDNA fragments have revealed that *LKB1* is ubiquitously expressed in fetal and adult tissues.^28^ Despite its ubiquitous expression, *LKB1* is alternatively spliced in different tissues, which may contribute to its function and, in some contexts, its pathology.^147^ After designing antibodies specific to N-terminal epitopes of LKB1, co-immunoprecipitation and immunoblotting revealed two protein bands in some tissues.^148^ As a result of variations in exon 9 splicing, several tissues produce both 50 kDa and 48 kDa long and short LKB1 isoforms, respectively.^147^ Both isoforms are widely expressed in humans, but most tissues preferentially express the 433 amino acid long isoform whereas the 404 amino acid short isoform is predominantly expressed in the testis.^149^^,^^150^ Compared with the LKB1 long isoform, the short isoform lacks 63 C-terminal residues, which are replaced by a 39-residue sequence^148^ (Fig. 2D). When *in vitro* kinase assays assessed LKB1 long and short isoform activity, there were no observable differences in LKB1-dependent kinase activation. Albeit, differences are observed in *in vivo* models.^148^^,^^149^^,^^151^ Male mice lacking the short LKB1 isoform displayed irregular spermatogenesis and were sterile whereas female mice lacking the short LKB1 isoform were fertile.^148^^,^^151^ Functional differences between LKB1 splice variants are not specific to mammals as *Drosophila melanogaster* expresses two different *LKB1* mRNAs where only the mRNA with a longer 5′ untranslated region is essential to spermatid morphogenesis.^149^

In addition to the LKB1 long and short splice isoforms and splice mutants implicated in PJS,^35^^,^^143^ alternative splicing of exon 1 is observed in a human lung cancer cell line, NCI-H460. NCI-H460 cells express a 42 kDa LKB1 isoform (ΔN-LKB1) 312 amino acids in length missing the N-terminal nuclear localization signal and part of the kinase domain (Fig. 2D).^12^ Although the kinase domain is incomplete, ΔN-LKB1 is restricted to the cytoplasm, increases the activity of downstream kinases, and functions in opposition to tumor suppressing wild-type LKB1. As such, inhibiting ΔN-LKB1 decreases the survival of NCI-H460 cells engrafted in nude mice.^12^ In addition to ΔN-LKB1, next generation sequencing technology has detected nonsense, frameshift, and splice mutations in exon 1 of *LKB1* across a broad scope of tumors.^152^ Given that deleting the splicing regulator *Rbm10* in *LKB1*-deficient mice had a moderate tumor suppressing effect,^153^ *LKB1* splicing may prove to be a suitable target to antagonize tumor cell survival.^154^

## Considerations prior to targeting LKB1 activity

### Variations in LKB1 splicing

A decade passed between characterizing *LKB1* splice mutants in PJS patients and identifying physiological *LKB1* splice variants^1^^,^^148^ and yet, despite success in detecting LKB1 splice sites,^155^ challenges detecting splice variants using genomic studies and computational algorithms remain.^156^ This is due to the numerous processes that regulate *LKB1* splicing. Indeed, mutations, cell and tissue types, species, and the species sex impact *LKB1* splicing and thus splicing is specific to each experimental model. Caution must be warranted when selecting an experimental model because the impact of alternative *LKB1* splice variants, if left undetected or omitted, may have drastic consequences on cell physiology and tumor pathology.^139^ Furthermore, early investigations raised LKB1 antibodies against the C-terminus,^157^ but based on the current understanding of LKB1 splice mutants, this is problematic as these antibodies only anneal to the long LKB1 isoform. Therefore, many historical investigations of LKB1 biology missed splicing variants, which may have drastically changed the outcomes regarding the role of LKB1 in physiology and disease.

### The impact of STRAD and MO25 on LKB1 activity

LKB1 catalytic activity depends on the formation of a 1:1:1 heterotrimeric complex *in vivo*, with the pseudokinase STRAD and mouse protein 25 (MO25) where mutations in all these proteins have proven to disrupt LKB1-STRAD-MO25 activity.^158^ In addition to mutations, miR-195 and miR-451 disrupt LKB1 signaling by targeting MO25.^134^ Therefore, steady-state levels of MO25 regulate the functional status of LKB1 and is thus important to LKB1 biology and pathology. Alternatively, there have been numerous investigations focused on linking STRAD to pathology. Given that *LKB1* is directly linked to the majority of PJS cases, it was hypothesized that *STRAD* may be a second genetic locus implicated in the remaining PJS cases.^159^ Thus, a loss-of-heterozygosity analysis screened for the presence of *STRAD* genetic deletions in PJS patients. In some cases, loss of heterozygosity was identified in a molecular marker near *STRAD*, yet sequencing discerned that all exons were intact suggesting STRAD is not linked to PJS.^160^ However, after isolating several STRADɑ splice variants in colorectal cancer cell lines, the interest of *STRAD* splice variants in tumorigenesis is renewed. In fact, measuring the activity of purified LKB1 complexes using LKB1tide kinase assays revealed that each STRADɑ splice variant differentially impact LKB1 localization, kinase activity, and downstream substrate activation.^161^ Thus, altering *STRADA* splicing may fine tune LKB1 signaling in tumorigenesis and serve as a potential therapeutic target.

Disrupting LKB1 activity may be achieved through several mechanisms including blocking LKB1 kinase activity, nucleocytoplasmic shuttling, and STRAD binding. Since STRAD binding is essential to LKB1 localization and protein stability *in vivo*,^162^ STRAD allosteric inhibitors are in development to disrupt LKB1 signaling.^163^ In fact, due to LKB1-independent STRAD activities, blocking STRAD instead of LKB1 may be more favorable. For instance, in LKB1-deficient lung cancer cells, STRAD facilitates cell invasion.^164^ Therefore, blocking LKB1 activity may increase LKB1-independent STRAD signaling which may facilitate invasion.

### The targeted delivery of LKB1 inhibitors

Although there is therapeutic merit in targeting LKB1 in cancer, there are some drawbacks delaying LKB1 inhibitor synthesis. First, LKB1 has historically been considered a tumor suppressor which could make targeting LKB1 in cancer treatment undesirable.^165^ Another limitation is the targeted delivery of LKB1 inhibitors as there is risk of spontaneous tumor formation in non-cancerous tissues relying on LKB1.^166^ For this reason, the ideal LKB1 inhibitor should be a small molecule signal transduction inhibitor capable of transversing across plasma and nuclear membranes specifically targeting LKB1 in tumor cells. Targeted therapies may be classified as “active” or “passive”. Active targeting involves inhibitors designed to target specific markers up-regulated in diseased cells whereas passive targeting combines inhibitors with nanoparticles to enhance inhibitor accumulation at the target.^167^ Since LKB1 is ubiquitously expressed in both non-cancerous and cancerous cells, LKB1 inhibitors would rely on passive targeting. As such, prior to generating LKB1 inhibitors, more research is needed to limit off-target effects, which is a critical component for feasible clinical application. One option when designing LKB1 inhibitors is to utilize liposome nanocarriers to improve the delivery of LKB1 inhibitors to tumors. Mechanistically, liposomes are designed to utilize properties of the tumor microenvironment, such as the leaky tumor vasculature, that enhance permeability and retention in tumor microenvironments.^168^ Other advantages of utilizing liposomes as the drug delivery system include the interaction between liposome and cell plasma membranes allowing direct release of inhibitors in target cells. Furthermore, the risk of patient adverse reactions is minimal due to the non-immunogenic, non-toxic, and biodegradable nature of liposomes.^169^ Due to advancements in drug delivery systems to enhance targeting specificity, there are many prospective delivery methodologies that mitigate risks associated with LKB1 inactivation in non-cancerous tissues.

## Targeting LKB1 activity

### Pharmacological agents targeting the LKB1-AMPK pathway

As previously discussed, the tumor suppressing functions of LKB1 are derived from screening tumor biopsies and conditional *in vivo* knockout models. These investigations demonstrate that there are numerous neoplasms with mutations in the LKB1-AMPK signaling pathway^46^ and these mutations are sufficient for spontaneous tumor formation in translational animal models.^170^, ^171^, ^172^ As such, pharmaceutically activating LKB1-AMPK signaling using AMPK agonists, such as metformin and 5-aminoimidazole-4-carboxamide ribonucleotide (AICAR), are under investigation as a potential therapeutic strategy for tumorigenesis. Unlike metformin and AICAR, A-769662, a small molecule AMPK agonist,^173^ increased tumor cell proliferation under low nutrient conditions.^174^ As such, despite extensive evidence linking LKB1 and AMPK to tumor suppressing processes, investigations implicating these proteins in tumor cell survival and metastasis are growing. Indeed, most screens of tumor biopsies report a shorter median overall survival for patients with *LKB1* mutant tumors citing increased disease progression, reduced time to treatment failure, and increased treatment failure frequency.^4^^,^^152^^,^^175^, ^176^, ^177^, ^178^, ^179^

Metformin activates AMPK through two mechanisms. The first relies on increasing AMP levels responsible for both activating and sustaining AMPK phosphorylation while the second involves promoting S428 LKB1 phosphorylation, which initiates LKB1 nuclear export and AMPK binding.^180^^,^^181^ The therapeutic potential of metformin is highlighted by its success in treating type 2 diabetes and reducing cancer mortality in type 2 diabetic patients by 57%.^182^ In addition to lowering cancer mortality in diabetic patients, meta-analyses of metformin clinical trials indicate that overall survival for a variety of cancers is improved.^183^, ^184^, ^185^ Similar to metformin, AICAR disrupts tumor migration, induces apoptosis,^15^ and has decreased lymphadenopathy in chronic lymphocyte leukemia clinical trials.^186^ The anti-tumorigenic properties of metformin are attributed to *in vitro* investigations finding both fewer tumor cells and cells lacking the ability to migrate in the presence of metformin.^187^^,^^188^ Alternatively, AICAR is metabolized to an AMP mimetic that binds to AMPKγ, which ultimately activates AMPK and disrupts mTOR.^16^ LKB1-AMPK activation has also suppressed tumor growth and delayed tumor onset in *PTEN*-deficient mice. A possible explanation is that during *PTEN* deficiency, the mTOR kinase is hyperactivated to augment tumorigenesis and facilitate therapeutic resistance. Therefore, disrupting mTOR by up-regulating LKB1-AMPK signaling is an emerging tumor therapeutic strategy.^189^

**Figure 3:**
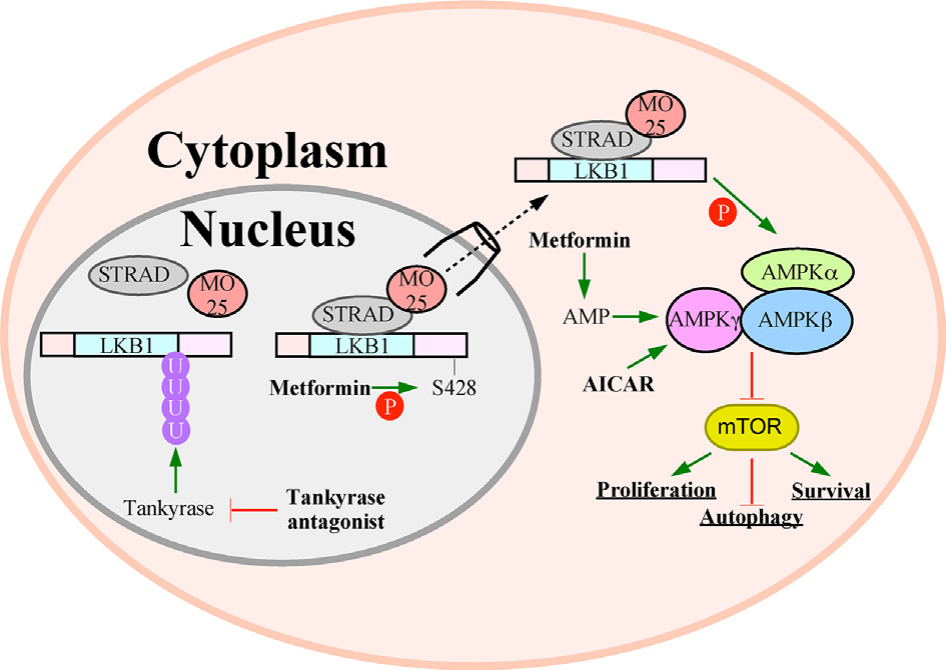
Tumor suppressing pathways of LKB1. The LKB1-AMPK-mTOR pathway suppresses tumor development. LKB1-AMPK disrupt mTOR activity leading to decreased survival and proliferation while increasing autophagy. Pharmacological compounds that activate the LKB1-AMPK axis and are proven to suppress tumorigenesis include a tankyrase antagonist, metformin, and 5-aminoimidazole-4-carboxamide ribonucleotide (AICAR). Tankyrase antagonists enhance LKB1-STRAD-MO25 complex forming by blocking tankyrase-dependent ubiquitination (U) of LKB1. Metformin increases S428 LKB1 phosphorylation and AMP levels both promoting AMPK activation. AICAR also functions as an AMP mimetic to increase AMPK activity.

Although clinical success has justified activating LKB1-AMPK signaling, there are several limitations regarding the use of metformin and AICAR as cancer therapeutics. First, due to poor oral bioavailability^190^ and renal toxicity,^191^ the therapeutic potential of AICAR is limited. Another limitation is the ambiguity of their mechanisms of action. Indeed, it is difficult to differentiate the AMPK-dependent and -independent effects as well as the role of LKB1.^192^ In fact, there is evidence suggesting that AICAR activates AMPK through LKB1-independent mechanisms.^193^ Therefore, the extent that the therapeutic properties may be attributed to LKB1-dependent AMPK activation is unknown. What is known is the therapeutic benefit of activating LKB1-AMPK has been replicated using agents that indirectly target the LKB1-AMPK axis. For instance, tankyrase inhibitors reduced mice lung tumor burden through increasing LKB1-AMPK activity as tankyrase1/2-dependent polyubiquitination of LKB1 disrupts LKB1-STRAD-MO25 complex formation (Fig. 3).^84^

### The tumor promoting properties of LKB1

Despite the success of LKB1-AMPK agonists, *LKB1* inactivation also increase the median overall survival for some tumor types.^194^, ^195^, ^196^, ^197^, ^198^, ^199^, ^200^, ^201^, ^202^, ^203^ Given that LKB1 activity up-regulates stress response pathways and ROS scavenging in tumor cells, LKB1 deficient tumors are more susceptible to metabolic stressors. Mechanistically, LKB1 inactivation increases ROS levels making tumor cells more susceptible to ROS-mediated cytotoxicity. In fact, cell lines lacking LKB1 are more susceptible to oxidative stress-inducing therapies such as cisplatin and γ-irradiation.^204^ Furthermore, LKB1-deficient NSCLC cell lines were more sensitive to tunicamycin and other endoplasmic reticulum stress activators.^205^ Patient derived xenografts generated from LKB1-deficient lung cancer patients displayed increased tumor necrosis with an impaired ability to adapt to metabolic stress mediated by the anti-angiogenic vascular endothelial growth factor (VEGF) inhibitor bevacizumab.^206^ In LKB1-deficient cells, chemical inhibitors that mediate metabolic stress including erlotinib and metformin have enhanced selectivity and cytotoxicity.^207^, ^208^, ^209^ The efficacy of poly(ADP-ribose) polymerase (PARP) inhibitors,^210^ extracellular signal-regulated kinase (ERK) inhibitors,^211^ and biguanide treatments^136^ are also improved when LKB1 expression is down-regulated. For these reasons, screening patients for LKB1 deficiency may dictate treatment combinations to improve patient survival while inactivating LKB1 in tumors enhances the efficacy of other cytotoxic compounds. Therefore, investigations should explore synthesizing novel LKB1 inhibitors as anti-tumorigenic agents.

A possible explanation of the pro-tumorigenic properties of the LKB1-AMPK axis involves mediating resistance to death via basement membrane detachment. AMPK protects tumor cells from anoikis through suppression of protein synthesis via mTOR inhibition.^67^ For instance, *in vitro* spheroid models of ovarian cancer and breast cancer demonstrated that *LKB1* expression is essential for tumor cell growth in suspension. Both CRISPR/Cas9-dependent *LKB1* knockout in ovarian cancer cells and *LKB1*-specific siRNA silencing in breast cancer cells decreased tumor burden and metastatic potential.^116^^,^^212^ However, the LKB1 effects in the ovarian cancer model were due to AMPK-independent mechanisms.^116^ Another potential explanation for pro-tumorigenic LKB1-AMPK signaling is disrupting NADPH consuming processes such as fatty acid synthesis while promoting NADPH producing processing like fatty acid oxidation.^66^ Since NADPH is essential to ROS scavenging, maintaining NADPH levels prolongs tumor cell survival in response to oxidative and metabolic stressors.^213^

**Figure 4:**
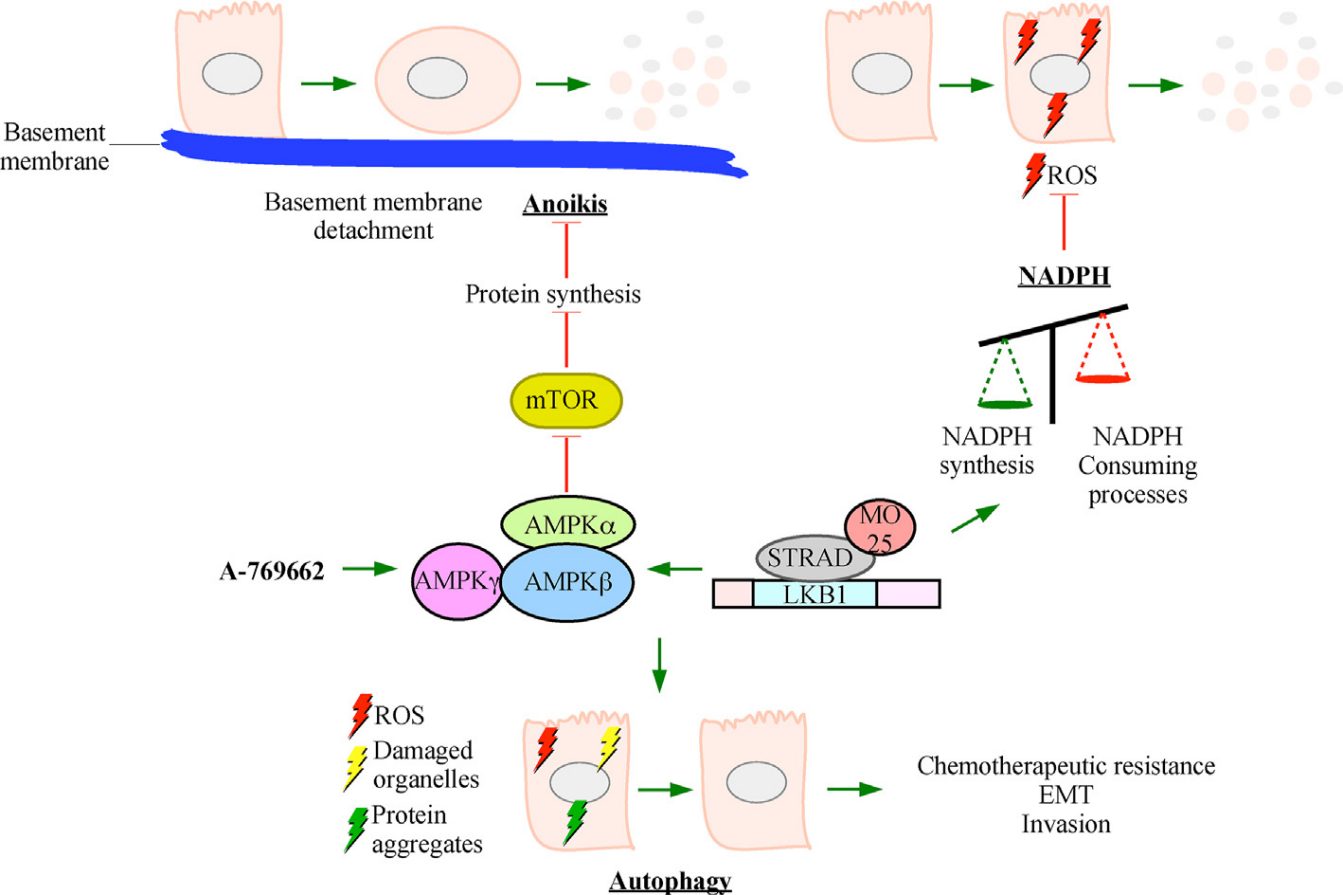
Tumor promoting roles of LKB1. LKB1-STRAD-MO25 activity can promote tumorigenesis by increasing reactive oxygen species (ROS) scavenging NADPH. Given that ROS damage tumor cells, increasing NADPH in tumors protects these cells from ROS-mediated damages. LKB1-AMPK signaling can enhance autophagy. Increasing autophagy in tumor cells leads to chemotherapeutic resistance, epithelial–mesenchymal transition (EMT), and invasion. Activating the LKB1-AMPK-mTOR pathway could protect tumor cells from anoikis, which is a form of cell death that occurs when cells detach from the basement membrane. A-769662 is an AMPK agonist that has increased tumorigenesis.

Protection against anoikis and ROS represent significant physiological benefits and justifies why some tumors increase the LKB1-AMPK pathway. Indeed, hepatocellular carcinoma adapts to energy stress by activating LKB1 through skp2-dependent K63 polyubiquitination.^13^ In addition to anoikis resistance and ROS scavenging, LKB1-AMPK up-regulates tumor metabolism. Tumor cells undergo extensive metabolic reprograming to adapt to their energy needs as many tumors switch from oxidation phosphorylation to aerobic glycolysis—known as the Warburg effect.^214^ In some instances, activating LKB1-AMPK in gastric cancer induced a metabolic shift that reversed the pro-tumorigenic Warburg effect^215^; however, LKB1-AMPK activate autophagy in response to metabolic and oxidative stress thus protecting cells from damage and apoptosis.^216^ Given that spontaneous tumors form in autophagy-related gene knockout models,^217^ autophagy suppresses tumor formation. Alternatively, tumors increase autophagic flux to protect against stress, chemotherapeutic agents, and promote invasion.^218^, ^219^, ^220^ Therefore, autophagy activation may be important for the tumor promoting properties of LKB1-AMPK signaling (Fig. 4).

## Concluding remarks

Genomic analyses first detected *LKB1* inactivation in pathology and recent improvements to these analyses and the development of next generation sequencing technologies pave the way for PJS and cancer diagnosis. For this reason, genomic medicine is the future of personalized therapy for pathologies induced by *LKB1* mutations.^43^^,^^221^ However, since LKB1 activity is regulated by protein–protein interactions, lipid binding, post-translational modifications, alternative splicing, and cellular localization, investigating LKB1 on pathology is currently beyond the scope of genomic studies alone. As such, advancements in *in silico* investigative practices, computational algorithms, and molecular techniques are together discerning the role of LKB1 in biology and oncology.

It is important to further explore context-specific roles for LKB1 and its regulation, especially given its paradoxical function in cancer. Prior to the recognition of its pro-tumorigeneic signaling, LKB1 was long regarded as strictly a tumor suppressor. However, the discovery of pro-tumorigenic LKB1 signaling combined with LKB1-deficiency enhancing the efficacy of other chemical agents, there is now a need to synthesize LKB1-specific inhibitors. Although there are now some inhibitors that indirectly target LKB1 signaling, there are no known pharmacological compounds that specifically antagonize LKB1.

Despite the extensive effort from numerous investigators providing unique perspectives, significant work is still warranted as few studies characterize how their findings function in context with other LKB1 regulating processes. If LKB1 is to be used as a disease biomarker, the appropriate tools and methods must be implemented to differentiate expression versus functional status. Therefore, the goal of this work was to highlight LKB1 activity in physiology and oncology providing a rationale for targeting LKB1 in cancer.

## Author contributions

Charles B. Trelford generated the first draft of the manuscript and generated the figures. Trevor G. Shepherd edited the manuscript and verified its scientific accuracy.

## Data availability

The data for Fig. 1 was obtained using cBioPortal for Cancer Genomics of the pan cancer study entitled MSK MetTropism (MSK, *Cell* 2021).

## Conflict of interests

None to disclose.

## References

[1] Hemminki A., Markie D., Tomlinson I. (1998). A serine/threonine kinase gene defective in Peutz-Jeghers syndrome. Nature.

[2] Li C., Lyu J., Meng Q.H. (2017). MiR-93 promotes tumorigenesis and metastasis of non-small cell lung cancer cells by activating the PI3K/Akt pathway via inhibition of *LKB1/PTEN/CDKN1A*. J Cancer.

[3] Matsumoto S., Iwakawa R., Takahashi K. (2007). Prevalence and specificity of LKB1 genetic alterations in lung cancers. Oncogene.

[4] Wingo S.N., Gallardo T.D., Akbay E.A. (2009). Somatic LKB1 mutations promote cervical cancer progression. PLoS One.

[5] Pierce S.E., Granja J.M., Corces M.R. (2021). LKB1 inactivation modulates chromatin accessibility to drive metastatic progression. Nat Cell Biol.

[6] McCarthy A., Lord C.J., Savage K. (2009). Conditional deletion of the Lkb1 gene in the mouse mammary gland induces tumour formation. J Pathol.

[7] Collet L., Ghurburrun E., Meyers N. (2020). *Kras* and *Lkb1* mutations synergistically induce intraductal papillary mucinous neoplasm derived from pancreatic duct cells. Gut.

[8] Zhuang Z.G., Di G.H., Shen Z.Z., Ding J., Shao Z.M. (2006). Enhanced expression of LKB1 in breast cancer cells attenuates angiogenesis, invasion, and metastatic potential. Mol Cancer Res.

[9] Qiu B., Wei W., Zhu J., Fu G., Lu D. (2018). EMT induced by loss of LKB1 promotes migration and invasion of liver cancer cells through ZEB1-induced YAP signaling. Oncol Lett.

[10] Liang X., Nan K.J., Li Z.L., Xu Q.Z. (2009). Overexpression of the LKB1 gene inhibits lung carcinoma cell proliferation partly through degradation of c-myc protein. Oncol Rep.

[11] Peart T., Ramos Valdes Y., Correa R.J. (2015). Intact LKB1 activity is required for survival of dormant ovarian cancer spheroids. Oncotarget.

[12] Dahmani R., Just P.A., Delay A. (2015). A novel LKB1 isoform enhances AMPK metabolic activity and displays oncogenic properties. Oncogene.

[13] Lee S.W., Li C.F., Jin G. (2015). Skp2-dependent ubiquitination and activation of LKB1 is essential for cancer cell survival under energy stress. Mol Cell.

[14] Hao B., Xiao Y., Song F. (2018). Metformin-induced activation of AMPK inhibits the proliferation and migration of human aortic smooth muscle cells through upregulation of p53 and IFI16. Int J Mol Med.

[15] Su C.C., Hsieh K.L., Liu P.L. (2019). AICAR induces apoptosis and inhibits migration and invasion in prostate cancer cells through an AMPK/mTOR-dependent pathway. Int J Mol Sci.

[16] Kong L., Zhang H., Lu C. (2021). AICAR, an AMP-activated protein kinase activator, ameliorates acute pancreatitis-associated liver injury partially through Nrf2-mediated antioxidant effects and inhibition of NLRP3 inflammasome activation. Front Pharmacol.

[17] Moro M., Caiola E., Ganzinelli M. (2018). Metformin enhances cisplatin-induced apoptosis and prevents resistance to cisplatin in co-mutated KRAS/LKB1 NSCLC. J Thorac Oncol.

[18] Gámez B., Morris E.V., Olechnowicz S.W.Z. (2022). The antidiabetic drug metformin acts on the bone microenvironment to promote myeloma cell adhesion to preosteoblasts and increase myeloma tumour burden *in vivo*. Transl Oncol.

[19] Sid B., Glorieux C., Valenzuela M. (2014). AICAR induces Nrf2 activation by an AMPK-independent mechanism in hepatocarcinoma cells. Biochem Pharmacol.

[20] Yamaguchi S., Kaneko M., Narukawa M. (2021). Approval success rates of drug candidates based on target, action, modality, application, and their combinations. Clin Transl Sci.

[21] Jeghers H., McKusick V.A., Katz K.H. (1949). Generalized intestinal polyposis and melanin spots of the oral mucosa, lips and digits. N Engl J Med.

[22] Foley T.R., McGarrity T.J., Abt A.B. (1988). Peutz-jeghers syndrome: a clinicopathologic survey of the "harrisburg family" with a 49-year follow-up. Gastroenterology.

[23] Peutz J.L. (1921). On a very remarkable case of familial polyposis of mucous membrane of intestinal tract and nasopharynx accompanied by peculiar pigmentations of skin and mucous membrane. Ned Tijdschr Geneeskd.

[24] Dib C., Fauré S., Fizames C. (1996). A comprehensive genetic map of the human genome based on 5, 264 microsatellites. Nature.

[25] Hemminki A., Tomlinson I., Markie D. (1997). Localization of a susceptibility locus for Peutz-Jeghers syndrome to 19p using comparative genomic hybridization and targeted linkage analysis. Nat Genet.

[26] Ylikorkala A., Avizienyte E., Tomlinson I.P. (1999). Mutations and impaired function of LKB1 in familial and non-familial Peutz-Jeghers syndrome and a sporadic testicular cancer. Hum Mol Genet.

[27] Avizienyte E., Roth S., Loukola A. (1998). Somatic mutations in LKB1 are rare in sporadic colorectal and testicular tumors. Cancer Res.

[28] Jenne D.E., Reimann H., Nezu J. (1998). Peutz-Jeghers syndrome is caused by mutations in a novel serine threonine kinase. Nat Genet.

[29] Boudeau J., Sapkota G., Alessi D.R. (2003). LKB1, a protein kinase regulating cell proliferation and polarity. FEBS Lett.

[30] Klein D. (2002). Quantification using real-time PCR technology: applications and limitations. Trends Mol Med.

[31] Olschwang S., Boisson C., Thomas G. (2001). Peutz-Jeghers families unlinked to STK11/LKB1 gene mutations are highly predisposed to primitive biliary adenocarcinoma. J Med Genet.

[32] Mehenni H., Blouin J.L., Radhakrishna U. (1997). Peutz-Jeghers syndrome: confirmation of linkage to chromosome 19p13.3 and identification of a potential second locus, on 19q13.4. Am J Hum Genet.

[33] Ashley D.J. (1969). The two “hit” and multiple “hit” theories of carcinogenesis. Br J Cancer.

[34] Tavusbay C., Acar T., Kar H., Atahan K., Kamer E. (2018). The patients with Peutz-Jeghers syndrome have a high risk of developing cancer. Turk J Surg.

[35] Su G.H., Hruban R.H., Bansal R.K. (1999). Germline and somatic mutations of the STK11/LKB1 Peutz-Jeghers gene in pancreatic and biliary cancers. Am J Pathol.

[36] Lin C., Lin X., Lin K., Tan J., Wei C., Liu T. (2021). LKB1 expression and the prognosis of lung cancer: a meta-analysis. Medicine.

[37] Kim C.J., Cho Y.G., Park J.Y. (2004). Genetic analysis of the LKB1/*STK11* gene in hepatocellular carcinomas. Eur J Cancer.

[38] Kenanli E., Karaman E., Enver O., Ulutin T., Buyru N. (2010). Genetic alterations of the *LKB1* gene in head and neck cancer. DNA Cell Biol.

[39] Miyaki M., Iijima T., Hosono K. (2000). Somatic mutations of LKB1 and beta-catenin genes in gastrointestinal polyps from patients with Peutz-Jeghers syndrome. Cancer Res.

[40] Nakanishi C., Yamaguchi T., Iijima T. (2004). Germline mutation of the LKB1/*STK11* gene with loss of the normal allele in an aggressive breast cancer of Peutz-Jeghers syndrome. Oncology.

[41] Nguyen B., Fong C., Luthra A. (2022). Genomic characterization of metastatic patterns from prospective clinical sequencing of 25, 000 patients. Cell.

[42] Cancer Genome Atlas Research Network (2012). Comprehensive genomic characterization of squamous cell lung cancers. Nature.

[43] Carretero J., Shimamura T., Rikova K. (2010). Integrative genomic and proteomic analyses identify targets for Lkb1-deficient metastatic lung tumors. Cancer Cell.

[44] Ding L., Getz G., Wheeler D.A. (2008). Somatic mutations affect key pathways in lung adenocarcinoma. Nature.

[45] Kottakis F., Nicolay B.N., Roumane A. (2016). LKB1 loss links serine metabolism to DNA methylation and tumorigenesis. Nature.

[46] Caiola E., Falcetta F., Giordano S. (2018). Co-occurring KRAS mutation/LKB1 loss in non-small cell lung cancer cells results in enhanced metabolic activity susceptible to caloric restriction: an *in vitro* integrated multilevel approach. J Exp Clin Cancer Res.

[47] Bange E., Marmarelis M.E., Hwang W.T. (2019). Impact of *KRAS* and *TP53* co-mutations on outcomes after first-line systemic therapy among patients with *STK11*-mutated advanced non-small-cell lung cancer. JCO Precis Oncol.

[48] Papillon-Cavanagh S., Doshi P., Dobrin R., Szustakowski J., Walsh A.M. (2020). *STK11* and *KEAP1* mutations as prognostic biomarkers in an observational real-world lung adenocarcinoma cohort. ESMO Open.

[49] Kitajima S., Ivanova E., Guo S. (2019). Suppression of STING associated with LKB1 loss in KRAS-driven lung cancer. Cancer Discov.

[50] Woods A., Johnstone S.R., Dickerson K. (2003). LKB1 is the upstream kinase in the AMP-activated protein kinase cascade. Curr Biol.

[51] Bright N.J., Thornton C., Carling D. (2009). The regulation and function of mammalian AMPK-related kinases. Acta Physiol.

[52] Shaw R.J., Kosmatka M., Bardeesy N. (2004). The tumor suppressor LKB1 kinase directly activates AMP-activated kinase and regulates apoptosis in response to energy stress. Proc Natl Acad Sci U S A..

[53] Maillet V., Boussetta N., Leclerc J. (2018). LKB1 as a gatekeeper of hepatocyte proliferation and genomic integrity during liver regeneration. Cell Rep.

[54] Patel K., Foretz M., Marion A. (2014). The LKB1-salt-inducible kinase pathway functions as a key gluconeogenic suppressor in the liver. Nat Commun.

[55] Shan T., Xiong Y., Kuang S. (2016). Deletion of Lkb1 in adult mice results in body weight reduction and lethality. Sci Rep.

[56] Barnes A.P., Lilley B.N., Pan Y.A. (2007). LKB1 and SAD kinases define a pathway required for the polarization of cortical neurons. Cell.

[57] Yamada E., Pessin J.E., Kurland I.J., Schwartz G.J., Bastie C.C. (2010). Fyn-dependent regulation of energy expenditure and body weight is mediated by tyrosine phosphorylation of LKB1. Cell Metabol.

[58] Tang H.M.V., Gao W.W., Chan C.P. (2013). LKB1 tumor suppressor and salt-inducible kinases negatively regulate human T-cell leukemia virus type 1 transcription. Retrovirology.

[59] Wang Y.S., Chen J., Cui F. (2016). LKB1 is a DNA damage response protein that regulates cellular sensitivity to PARP inhibitors. Oncotarget.

[60] Karuman P., Gozani O., Odze R.D. (2001). The Peutz-Jegher gene product LKB1 is a mediator of p53-dependent cell death. Mol Cell.

[61] Shackelford D.B., Shaw R.J. (2009). The LKB1-AMPK pathway: metabolism and growth control in tumour suppression. Nat Rev Cancer.

[62] Carretero J., Medina P.P., Blanco R. (2007). Dysfunctional AMPK activity, signalling through mTOR and survival in response to energetic stress in LKB1-deficient lung cancer. Oncogene.

[63] Huang X., Wullschleger S., Shpiro N. (2008). Important role of the LKB1-AMPK pathway in suppressing tumorigenesis in PTEN-deficient mice. Biochem J.

[64] Feng Y., Yang J., Wang Y. (2024). Cafestol inhibits colon cancer cell proliferation and tumor growth in xenograft mice by activating LKB1/AMPK/ULK1-dependent autophagy. J Nutr Biochem.

[65] Hou T., Wang Y., Dan W. (2023). β-Ionone represses renal cell carcinoma progression through activating LKB1/AMPK-triggered autophagy. J Biochem Mol Toxicol.

[66] Lin R., Elf S., Shan C. (2015). 6-Phosphogluconate dehydrogenase links oxidative PPP, lipogenesis and tumour growth by inhibiting LKB1-AMPK signalling. Nat Cell Biol.

[67] Ng T.L., Leprivier G., Robertson M.D. (2012). The AMPK stress response pathway mediates anoikis resistance through inhibition of mTOR and suppression of protein synthesis. Cell Death Differ.

[68] Casimiro M.C., Di Sante G., di Rocco A. (2017). Cyclin D1 restrains oncogene-induced autophagy by regulating the AMPK-LKB1 signaling axis. Cancer Res.

[69] Murray C.W., Brady J.J., Tsai M.K. (2019). An LKB1-SIK axis suppresses lung tumor growth and controls differentiation. Cancer Discov.

[70] Hollstein P.E., Eichner L.J., Brun S.N. (2019). The AMPK-related kinases SIK_1_ and SIK_3_ mediate key tumor-suppressive effects of LKB1 in NSCLC. Cancer Discov.

[71] Tarumoto Y., Lu B., Somerville T.D.D. (2018). LKB1, salt-inducible kinases, and MEF2C are linked dependencies in acute myeloid leukemia. Mol Cell.

[72] Zagórska A., Deak M., Campbell D.G. (2010). New roles for the LKB1-NUAK pathway in controlling myosin phosphatase complexes and cell adhesion. Sci Signal.

[73] Whyte D., Skalka G., Walsh P. (2023). NUAK1 governs centrosome replication in pancreatic cancer via MYPT1/PP1β and GSK3β-dependent regulation of PLK4. Mol Oncol.

[74] Machino H., Kaneko S., Komatsu M. (2022). The metabolic stress-activated checkpoint LKB1-MARK3 axis acts as a tumor suppressor in high-grade serous ovarian carcinoma. Commun Biol.

[75] Rines A.K., Burke M.A., Fernandez R.P., Volpert O.V., Ardehali H. (2012). Snf1-related kinase inhibits colon cancer cell proliferation through calcyclin-binding protein-dependent reduction of β-catenin. Faseb J.

[76] Fukuyama M., Sakuma K., Park R. (2012). C. elegans AMPKs promote survival and arrest germline development during nutrient stress. Biol Open.

[77] Bland M.L., Lee R.J., Magallanes J.M., Foskett J.K., Birnbaum M.J. (2010). AMPK supports growth in *Drosophila* by regulating muscle activity and nutrient uptake in the gut. Dev Biol.

[78] Ikeda Y., Sato K., Pimentel D.R. (2009). Cardiac-specific deletion of LKB1 leads to hypertrophy and dysfunction. J Biol Chem.

[79] Fu A., Robitaille K., Faubert B. (2015). LKB1 couples glucose metabolism to insulin secretion in mice. Diabetologia.

[80] Thomson D.M., Porter B.B., Tall J.H., Kim H.J., Barrow J.R., Winder W.W. (2007). Skeletal muscle and heart LKB1 deficiency causes decreased voluntary running and reduced muscle mitochondrial marker enzyme expression in mice. Am J Physiol Endocrinol Metab.

[81] Han S.H., Malaga-Dieguez L., Chinga F. (2016). Deletion of Lkb1 in renal tubular epithelial cells leads to CKD by altering metabolism. J Am Soc Nephrol.

[82] Xu Y., Gao Y., Huang Z. (2019). LKB1 suppresses androgen synthesis in a mouse model of hyperandrogenism via IGF-1 signaling. FEBS Open Bio.

[83] George S.H., Milea A., Sowamber R., Chehade R., Tone A., Shaw P.A. (2016). Loss of LKB1 and p53 synergizes to alter fallopian tube epithelial phenotype and high-grade serous tumorigenesis. Oncogene.

[84] Li N., Wang Y., Neri S. (2019). Tankyrase disrupts metabolic homeostasis and promotes tumorigenesis by inhibiting LKB1-AMPK signalling. Nat Commun.

[85] Contreras C.M., Akbay E.A., Gallardo T.D. (2010). Lkb1 inactivation is sufficient to drive endometrial cancers that are aggressive yet highly responsive to mTOR inhibitor monotherapy. Dis Model Mech.

[86] Tanwar P.S., Kaneko-Tarui T., Zhang L., Teixeira J.M. (2012). Altered LKB1/AMPK/TSC1/TSC2/mTOR signaling causes disruption of Sertoli cell polarity and spermatogenesis. Hum Mol Genet.

[87] Asada N., Sanada K., Fukada Y. (2007). LKB1 regulates neuronal migration and neuronal differentiation in the developing neocortex through centrosomal positioning. J Neurosci.

[88] Men Y., Zhang A., Li H. (2015). LKB1 regulates cerebellar development by controlling sonic hedgehog-mediated granule cell precursor proliferation and granule cell migration. Sci Rep.

[89] Asada N., Sanada K. (2010). LKB1-mediated spatial control of GSK3 and adenomatous polyposis coli contributes to centrosomal forward movement and neuronal migration in the developing neocortex. J Neurosci.

[90] Dasgupta B., Milbrandt J. (2009). AMP-activated protein kinase phosphorylates retinoblastoma protein to control mammalian brain development. Dev Cell.

[91] Williams T., Courchet J., Viollet B., Brenman J.E., Polleux F. (2011). AMP-activated protein kinase (AMPK) activity is not required for neuronal development but regulates axogenesis during metabolic stress. Proc Natl Acad Sci USA.

[92] Claret M., Smith M.A., Knauf C. (2011). Deletion of Lkb1 in pro-opiomelanocortin neurons impairs peripheral glucose homeostasis in mice. Diabetes.

[93] McClatchy D.B., Ma Y., Liu C. (2015). Pulsed azidohomoalanine labeling in mammals (PALM) detects changes in liver-specific LKB1 knockout mice. J Proteome Res.

[94] Shaw R.J., Lamia K.A., Vasquez D. (2005). The kinase LKB1 mediates glucose homeostasis in liver and therapeutic effects of metformin. Science.

[95] Mitsuhashi K., Senmaru T., Fukuda T. (2016). Testosterone stimulates glucose uptake and GLUT4 translocation through LKB1/AMPK signaling in 3T3-L1 adipocytes. Endocrine.

[96] Sakamoto K., McCarthy A., Smith D. (2005). Deficiency of LKB1 in skeletal muscle prevents AMPK activation and glucose uptake during contraction. EMBO J.

[97] Li Y., Xu S., Mihaylova M.M. (2011). AMPK phosphorylates and inhibits SREBP activity to attenuate hepatic steatosis and atherosclerosis in diet-induced insulin-resistant mice. Cell Metabol.

[98] Li C., Dong X., Du W. (2020). LKB1-AMPK axis negatively regulates ferroptosis by inhibiting fatty acid synthesis. Signal Transduct Targeted Ther.

[99] Mans L.A., Querol Cano L., van Pelt J., Giardoglou P., Keune W.J., Haramis A.G. (2017). The tumor suppressor LKB1 regulates starvation-induced autophagy under systemic metabolic stress. Sci Rep.

[100] Hezel A.F., Gurumurthy S., Granot Z. (2008). Pancreatic LKB1 deletion leads to acinar polarity defects and cystic neoplasms. Mol Cell Biol.

[101] Han Y., Feng H., Sun J. (2019). Lkb1 deletion in periosteal mesenchymal progenitors induces osteogenic tumors through mTORC1 activation. J Clin Investig.

[102] Nakau M., Miyoshi H., Seldin M.F., Imamura M., Oshima M., Taketo M.M. (2002). Hepatocellular carcinoma caused by loss of heterozygosity in Lkb1 gene knockout mice. Cancer Res.

[103] Shorning B.Y., Griffiths D., Clarke A.R. (2011). Lkb1 and Pten synergise to suppress mTOR-mediated tumorigenesis and epithelial-mesenchymal transition in the mouse bladder. PLoS One.

[104] Pearson H.B., McCarthy A., Collins C.M., Ashworth A., Clarke A.R. (2008). Lkb1 deficiency causes prostate neoplasia in the mouse. Cancer Res.

[105] Kou B., Kou Q., Ma B. (2018). Thymoquinone inhibits metastatic phenotype and epithelial-mesenchymal transition in renal cell carcinoma by regulating the LKB1/AMPK signaling pathway. Oncol Rep.

[106] Wang L., Li H., Zhen Z. (2019). CXCL17 promotes cell metastasis and inhibits autophagy via the LKB1-AMPK pathway in hepatocellular carcinoma. Gene.

[107] Kan J.Y., Yen M.C., Wang J.Y. (2016). Nesfatin-1/Nucleobindin-2 enhances cell migration, invasion, and epithelial-mesenchymal transition via LKB1/AMPK/TORC1/ZEB1 pathways in colon cancer. Oncotarget.

[108] Li N.S., Zou J.R., Lin H. (2016). LKB1/AMPK inhibits TGF-β1 production and the TGF-β signaling pathway in breast cancer cells. Tumour Biol.

[109] Li J., Liu J., Li P. (2014). Loss of LKB1 disrupts breast epithelial cell polarity and promotes breast cancer metastasis and invasion. J Exp Clin Cancer Res.

[110] Zheng X., Chi J., Zhi J. (2018). Aurora-A-mediated phosphorylation of LKB1 compromises LKB1/AMPK signaling axis to facilitate NSCLC growth and migration. Oncogene.

[111] Ji H., Ramsey M.R., Hayes D.N. (2007). LKB1 modulates lung cancer differentiation and metastasis. Nature.

[112] Han X., Li F., Fang Z. (2014). Transdifferentiation of lung adenocarcinoma in mice with Lkb1 deficiency to squamous cell carcinoma. Nat Commun.

[113] Tong X., Patel A.S., Kim E. (2024). Adeno-to-squamous transition drives resistance to KRAS inhibition in LKB1 mutant lung cancer. Cancer Cell.

[114] Xu C., Fillmore C.M., Koyama S. (2014). Loss of Lkb1 and Pten leads to lung squamous cell carcinoma with elevated PD-L1 expression. Cancer Cell.

[115] Kim E.K., Park J.M., Lim S. (2011). Activation of AMP-activated protein kinase is essential for lysophosphatidic acid-induced cell migration in ovarian cancer cells. J Biol Chem.

[116] Buensuceso A., Ramos-Valdes Y., DiMattia G.E., Shepherd T.G. (2020). AMPK-independent LKB1 activity is required for efficient epithelial ovarian cancer metastasis. Mol Cancer Res.

[117] Volik S., Lebedev Y., Nikolaev L. (1995). Mapping of transcribed sequences on human chromosome 19. DNA Sequence.

[118] Ashworth L.K., Batzer M.A., Brandriff B. (1995). An integrated metric physical map of human chromosome 19. Nat Genet.

[119] Nezu J. (1996).

[120] Yoneda Y. (1997). How proteins are transported from cytoplasm to the nucleus. J Biochem.

[121] Smith D.P., Spicer J., Smith A., Swift S., Ashworth A. (1999). The mouse Peutz-Jeghers syndrome gene Lkb1 encodes a nuclear protein kinase. Hum Mol Genet.

[122] Esteller M., Fraga M.F., Guo M. (2001). DNA methylation patterns in hereditary human cancers mimic sporadic tumorigenesis. Hum Mol Genet.

[123] Sun R., Li J., Wang B. (2015). Liver kinase B1 promoter CpG island methylation is related to lung cancer and smoking. Int J Clin Exp Med.

[124] Zheng F., Yuan X., Chen E., Ye Y., Li X., Dai Y. (2017). Methylation of *STK11* promoter is a risk factor for tumor stage and survival in clear cell renal cell carcinoma. Oncol Lett.

[125] Li T., Lin W., Zhao Y., Zhu J., Sun T., Ren L. (2020). Distinct promoter methylation patterns of LKB1 in the hamartomatous polyps of Peutz-Jeghers syndrome and its potential in gastrointestinal malignancy prediction. Orphanet J Rare Dis.

[126] Zhang W., Li X., Song G., Luo D. (2017). Prognostic significance of LKB1 promoter methylation in cutaneous malignant melanoma. Oncol Lett.

[127] Koenig M.J., Agana B.A., Kaufman J.M. (2021). STK11/LKB1 loss of function is associated with global DNA hypomethylation and *S*-adenosyl-methionine depletion in human lung adenocarcinoma. Cancer Res.

[128] Borzi C., Ganzinelli M., Caiola E. (2021). LKB1 down-modulation by miR-17 identifies patients with NSCLC having worse prognosis eligible for energy-stress-based treatments. J Thorac Oncol.

[129] Boldrini L., Giordano M., Lucchi M., Melfi F., Fontanini G. (2018). Expression profiling and microRNA regulation of the LKB1 pathway in young and aged lung adenocarcinoma patients. Biomed Rep.

[130] Avtanski D.B., Nagalingam A., Bonner M.Y., Arbiser J.L., Saxena N.K., Sharma D. (2015). Honokiol activates LKB1-miR-34a axis and antagonizes the oncogenic actions of leptin in breast cancer. Oncotarget.

[131] Figueroa-González G., Carrillo-Hernández J.F., Perez-Rodriguez I. (2020). Negative regulation of serine threonine kinase 11 (STK11) through miR-100 in head and neck cancer. Genes.

[132] Zhou Y., Zhang Y., Li Y. (2021). MicroRNA-106a-5p promotes the proliferation, autophagy and migration of lung adenocarcinoma cells by targeting LKB1/AMPK. Exp Ther Med.

[133] Fang X., Shen F., Lechauve C. (2018). *miR-144/451* represses the LKB1/AMPK/mTOR pathway to promote red cell precursor survival during recovery from acute anemia. Haematologica.

[134] Chen H., Untiveros G.M., McKee L.A. (2012). Micro-RNA-195 and -451 regulate the LKB1/AMPK signaling axis by targeting MO25. PLoS One.

[135] Godlewski J., Nowicki M.O., Bronisz A. (2010). MicroRNA-451 regulates LKB1/AMPK signaling and allows adaptation to metabolic stress in glioma cells. Mol Cell.

[136] Izreig S., Gariepy A., Kaymak I. (2020). Repression of LKB1 by *miR-17∼92* sensitizes *MYC*-dependent lymphoma to biguanide treatment. Cell Rep Med.

[137] Islam M.J., Khan A.M., Parves M.R., Hossain M.N., Halim M.A. (2019). Prediction of deleterious non-synonymous SNPs of human *STK11* gene by combining algorithms, molecular docking, and molecular dynamics simulation. Sci Rep.

[138] Radenbaugh A.J., Ma S., Ewing A. (2014). *RADIA*: RNA and DNA integrated analysis for somatic mutation detection. PLoS One.

[139] Ishqi H.M., Sarwar T., Husain M.A., Rehman S.U., Tabish M. (2018). Differentially expressed novel alternatively spliced transcript variant of tumor suppressor Stk11 gene in mouse. Gene.

[140] Lützner N., De-Castro Arce J., Rösl F. (2012). Gene expression of the tumour suppressor LKB1 is mediated by Sp1, NF-Y and FOXO transcription factors. PLoS One.

[141] Rabbani B., Mahdieh N., Haghi Ashtiani M.T., Setoodeh A., Rabbani A. (2012). In silico structural, functional and pathogenicity evaluation of a novel mutation: an overview of *HSD3B2* gene mutations. Gene.

[142] Lim W., Hearle N., Shah B. (2003). Further observations on LKB1/STK11 status and cancer risk in Peutz-Jeghers syndrome. Br J Cancer.

[143] Mehenni H., Gehrig C., Nezu J. (1998). Loss of LKB1 kinase activity in Peutz-Jeghers syndrome, and evidence for allelic and locus heterogeneity. Am J Hum Genet.

[144] Newton S.M., Igo J.D., Scott D.C., Klebba P.E. (1999). Effect of loop deletions on the binding and transport of ferric enterobactin by FepA. Mol Microbiol.

[145] Batey S., Nickson A.A., Clarke J. (2008). Studying the folding of multidomain proteins. HFSP J.

[146] Collins S.P., Reoma J.L., Gamm D.M., Uhler M.D. (2000). LKB1, a novel serine/threonine protein kinase and potential tumour suppressor, is phosphorylated by cAMP-dependent protein kinase (PKA) and prenylated *in vivo*. Biochem J.

[147] Denison F.C., Hiscock N.J., Carling D., Woods A. (2009). Characterization of an alternative splice variant of LKB1. J Biol Chem.

[148] Towler M.C., Fogarty S., Hawley S.A. (2008). A novel short splice variant of the tumour suppressor LKB1 is required for spermiogenesis. Biochem J.

[149] Couderc J.L., Richard G., Vachias C., Mirouse V. (2017). *Drosophila* LKB1 is required for the assembly of the polarized actin structure that allows spermatid individualization. PLoS One.

[150] Laderian B., Mundi P., Fojo T., Bates S.E. (2020). Emerging therapeutic implications of STK11 mutation: case series. Oncol.

[151] Kong F., Wang M., Huang X. (2017). Differential regulation of spermatogenic process by Lkb1 isoforms in mouse testis. Cell Death Dis.

[152] Pécuchet N., Laurent-Puig P., Mansuet-Lupo A. (2017). Different prognostic impact of STK11 mutations in non-squamous non-small-cell lung cancer. Oncotarget.

[153] Rogers Z.N., McFarland C.D., Winters I.P. (2018). Mapping the *in vivo* fitness landscape of lung adenocarcinoma tumor suppression in mice. Nat Genet.

[154] Momcilovic M., Shackelford D.B. (2015). Targeting LKB1 in cancer - exposing and exploiting vulnerabilities. Br J Cancer.

[155] Zhao N., Wu H., Li P. (2021). A novel pathogenic splice site variation in *STK11* gene results in Peutz-Jeghers syndrome. Mol Genet Genomic Med.

[156] Jian X., Boerwinkle E., Liu X. (2014). In silico prediction of splice-altering single nucleotide variants in the human genome. Nucleic Acids Res.

[157] Tiainen M., Ylikorkala A., Mäkelä T.P. (1999). Growth suppression by Lkb1 is mediated by a G_1_ cell cycle arrest. Proc Natl Acad Sci U S A.

[158] Baas A.F., Boudeau J., Sapkota G.P. (2003). Activation of the tumour suppressor kinase LKB1 by the STE20-like pseudokinase STRAD. EMBO J.

[159] Buchet-Poyau K., Mehenni H., Radhakrishna U., Antonarakis S.E. (2002). Search for the second Peutz-Jeghers syndrome locus: exclusion of the STK13, PRKCG, KLK10, and *PSCD2* genes on chromosome 19 and the *STK11IP* gene on chromosome 2. Cytogenet Genome Res.

[160] de Leng W.W., Keller J.J., Luiten S. (2005). STRAD in Peutz-Jeghers syndrome and sporadic cancers. J Clin Pathol.

[161] Marignani P.A., Scott K.D., Bagnulo R. (2007). Novel splice isoforms of STRADalpha differentially affect LKB1 activity, complex assembly and subcellular localization. Cancer Biol Ther.

[162] Dorfman J., Macara I.G. (2008). STRADα regulates LKB1 localization by blocking access to importin-α, and by association with Crm1 and exportin-7. MBoC.

[163] Qing T., Liu J., Liu F., Mitchell D.C., Beresis R.T., Gordan J.D. (2022). Methods to assess small molecule allosteric modulators of the STRAD pseudokinase. Methods Enzymol.

[164] Eggers C.M., Kline E.R., Zhong D., Zhou W., Marcus A.I. (2012). STE20-related kinase adaptor protein α (STRADα) regulates cell polarity and invasion through PAK1 signaling in LKB1-null cells. J Biol Chem.

[165] Duivenvoorden W.C., Beatty L.K., Lhotak S. (2013). Underexpression of tumour suppressor LKB1 in clear cell renal cell carcinoma is common and confers growth advantage *in vitro* and *in vivo*. Br J Cancer.

[166] Contreras C.M., Gurumurthy S., Haynie J.M. (2008). Loss of Lkb1 provokes highly invasive endometrial adenocarcinomas. Cancer Res.

[167] Adepu S., Ramakrishna S. (2021). Controlled drug delivery systems: current status and future directions. Molecules.

[168] Wang S., Chen Y., Guo J., Huang Q. (2023). Liposomes for tumor targeted therapy: a review. Int J Mol Sci.

[169] Guimarães D., Cavaco-Paulo A., Nogueira E. (2021). Design of liposomes as drug delivery system for therapeutic applications. Int J Pharm.

[170] Sanchez-Cespedes M., Parrella P., Esteller M. (2002). Inactivation of LKB1/STK11 is a common event in adenocarcinomas of the lung. Cancer Res.

[171] Mung K.L., Eccleshall W.B., Santio N.M., Rivero-Müller A., Koskinen P.J. (2021). PIM kinases inhibit AMPK activation and promote tumorigenicity by phosphorylating LKB1. Cell Commun Signal.

[172] Rao F., Xu J., Fu C. (2015). Inositol pyrophosphates promote tumor growth and metastasis by antagonizing liver kinase B1. Proc Natl Acad Sci U S A.

[173] Göransson O., McBride A., Hawley S.A. (2007). Mechanism of action of A-769662, a valuable tool for activation of AMP-activated protein kinase. J Biol Chem.

[174] Vincent E.E., Coelho P.P., Blagih J., Griss T., Viollet B., Jones R.G. (2015). Differential effects of AMPK agonists on cell growth and metabolism. Oncogene.

[175] Hirose S., Murakami N., Takahashi K. (2020). Genomic alterations in STK11 can predict clinical outcomes in cervical cancer patients. Gynecol Oncol.

[176] Selenica P., Alemar B., Matrai C. (2021). Massively parallel sequencing analysis of 68 gastric-type cervical adenocarcinomas reveals mutations in cell cycle-related genes and potentially targetable mutations. Mod Pathol.

[177] Facchinetti F., Bluthgen M.V., Tergemina-Clain G. (2017). LKB1/STK11 mutations in non-small cell lung cancer patients: descriptive analysis and prognostic value. Lung Cancer.

[178] Shire N.J., Klein A.B., Golozar A. (2020). STK11(LKB1) mutations in metastatic NSCLC: prognostic value in the real world. PLoS One.

[179] Rosellini P., Amintas S., Caumont C. (2022). Clinical impact of STK11 mutation in advanced-stage non-small cell lung cancer. Eur J Cancer.

[180] Dowling R.J., Zakikhani M., Fantus I.G., Pollak M., Sonenberg N. (2007). Metformin inhibits mammalian target of rapamycin-dependent translation initiation in breast cancer cells. Cancer Res.

[181] Xie Z., Dong Y., Scholz R., Neumann D., Zou M.H. (2008). Phosphorylation of LKB1 at serine 428 by protein kinase C-ζ is required for metformin-enhanced activation of the AMP-activated protein kinase in endothelial cells. Circulation.

[182] Landman G.W., Kleefstra N., van Hateren K.J., Groenier K.H., Gans R.O., Bilo H.J. (2010). Metformin associated with lower cancer mortality in type 2 diabetes. Diabetes Care.

[183] Hui T., Shang C., Yang L., Wang M., Li R., Song Z. (2021). Metformin improves the outcomes in Chinese invasive breast cancer patients with type 2 diabetes mellitus. Sci Rep.

[184] Meng F., Song L., Wang W. (2017). Metformin improves overall survival of colorectal cancer patients with diabetes: a meta-analysis. J Diabetes Res.

[185] Zeng S., Gan H.X., Xu J.X., Liu J.Y. (2019). Metformin improves survival in lung cancer patients with type 2 diabetes mellitus: a meta-analysis. Med Clínica Engl Ed.

[186] van Den Neste E., Cazin B., Janssens A. (2013). Acadesine for patients with relapsed/refractory chronic lymphocytic leukemia (CLL): a multicenter phase I/II study. Cancer Chemother Pharmacol.

[187] Bao B., Wang Z., Ali S. (2012). Metformin inhibits cell proliferation, migration and invasion by attenuating CSC function mediated by deregulating miRNAs in pancreatic cancer cells. Cancer Prev Res.

[188] Chen Y.H., Yang S.F., Yang C.K. (2021). Metformin induces apoptosis and inhibits migration by activating the AMPK/p53 axis and suppressing PI3K/AKT signaling in human cervical cancer cells. Mol Med Rep.

[189] Ilagan E., Manning B.D. (2016). Emerging role of mTOR in the response to cancer therapeutics. Trends Cancer.

[190] Dixon R., Gourzis J., McDermott D., Fujitaki J., Dewland P., Gruber H. (1991). AICA-riboside: safety, tolerance, and pharmacokinetics of a novel adenosine-regulating agent. J Clin Pharmacol.

[191] Cluzeau T., Furstoss N., Savy C. (2019). Acadesine circumvents azacitidine resistance in myelodysplastic syndrome and acute myeloid leukemia. Int J Mol Sci.

[192] Rena G., Hardie D.G., Pearson E.R. (2017). The mechanisms of action of metformin. Diabetologia.

[193] Sun Y., Connors K.E., Yang D.Q. (2007). AICAR induces phosphorylation of AMPK in an ATM-dependent, LKB1-independent manner. Mol Cell Biochem.

[194] Caso R., Sanchez-Vega F., Tan K.S. (2020). The underlying tumor genomics of predominant histologic subtypes in lung adenocarcinoma. J Thorac Oncol.

[195] Jee J., Lebow E.S., Yeh R. (2022). Overall survival with circulating tumor DNA-guided therapy in advanced non-small-cell lung cancer. Nat Med.

[196] Giraldo N.A., Drill E., Satravada B.A. (2022). Comprehensive molecular characterization of gallbladder carcinoma and potential targets for intervention. Clin Cancer Res.

[197] Elkin R., Oh J.H., Liu Y.L. (2021). Geometric network analysis provides prognostic information in patients with high grade serous carcinoma of the ovary treated with immune checkpoint inhibitors. NPJ Genom Med.

[198] Sanchez-Vega F., Mina M., Armenia J. (2018). Oncogenic signaling pathways in the cancer genome atlas. Cell.

[199] Liu D., Schilling B., Liu D. (2019). Integrative molecular and clinical modeling of clinical outcomes to PD1 blockade in patients with metastatic melanoma. Nat Med.

[200] Sihag S., Nussenzweig S.C., Walch H.S. (2022). The role of the TP53 pathway in predicting response to neoadjuvant therapy in esophageal adenocarcinoma. Clin Cancer Res.

[201] Yaeger R., Chatila W.K., Lipsyc M.D. (2018). Clinical sequencing defines the genomic landscape of metastatic colorectal cancer. Cancer Cell.

[202] Nacev B.A., Sanchez-Vega F., Smith S.A. (2022). Clinical sequencing of soft tissue and bone sarcomas delineates diverse genomic landscapes and potential therapeutic targets. Nat Commun.

[203] Ho A.S., Ochoa A., Jayakumaran G. (2019). Genetic hallmarks of recurrent/metastatic adenoid cystic carcinoma. J Clin Investig.

[204] Zulato E., Ciccarese F., Agnusdei V. (2018). LKB1 loss is associated with glutathione deficiency under oxidative stress and sensitivity of cancer cells to cytotoxic drugs and γ-irradiation. Biochem Pharmacol.

[205] Inge L.J., Friel J.M., Richer A.L. (2014). LKB1 inactivation sensitizes non-small cell lung cancer to pharmacological aggravation of ER stress. Cancer Lett.

[206] Bonanno L., de Paoli A., Zulato E. (2017). LKB1 expression correlates with increased survival in patients with advanced non-small cell lung cancer treated with chemotherapy and bevacizumab. Clin Cancer Res.

[207] Rho S.B., Byun H.J., Kim B.R., Lee C.H. (2021). Knockdown of *LKB1* sensitizes endometrial cancer cells via AMPK activation. Biomol Ther (Seoul).

[208] Shameem M., Bagherpoor A.J., Nakhi A., Dosa P., Georg G., Kassie F. (2023). Mitochondria-targeted metformin (mitomet) inhibits lung cancer in cellular models and in mice by enhancing the generation of reactive oxygen species. Mol Carcinog.

[209] Whang Y.M., Park S.I., Trenary I.A. (2016). LKB1 deficiency enhances sensitivity to energetic stress induced by erlotinib treatment in non-small-cell lung cancer (NSCLC) cells. Oncogene.

[210] Long L.L., Ma S.C., Guo Z.Q. (2023). PARP inhibition induces synthetic lethality and adaptive immunity in LKB1-mutant lung cancer. Cancer Res.

[211] Caiola E., Iezzi A., Tomanelli M. (2020). LKB1 deficiency renders NSCLC cells sensitive to ERK inhibitors. J Thorac Oncol.

[212] Trapp E.K., Majunke L., Zill B. (2017). LKB1 pro-oncogenic activity triggers cell survival in circulating tumor cells. Mol Oncol.

[213] Jeon S.M., Chandel N.S., Hay N. (2012). AMPK regulates NADPH homeostasis to promote tumour cell survival during energy stress. Nature.

[214] Jiang S., Wang Y., Luo L. (2019). AMP-activated protein kinase regulates cancer cell growth and metabolism via nuclear and mitochondria events. J Cell Mol Med.

[215] Li W., Wong C.C., Zhang X. (2018). CAB39L elicited an anti-Warburg effect via a LKB1-AMPK-PGC1α axis to inhibit gastric tumorigenesis. Oncogene.

[216] Zhang M., Deng Y.N., Zhang J.Y. (2018). SIRT3 protects rotenone-induced injury in SH-SY_5_Y cells by promoting autophagy through the LKB1-AMPK-mTOR pathway. Aging Dis.

[217] Takahashi Y., Coppola D., Matsushita N. (2007). Bif-1 interacts with beclin 1 through UVRAG and regulates autophagy and tumorigenesis. Nat Cell Biol.

[218] Trelford C.B., di Guglielmo G.M. (2021). Molecular mechanisms of mammalian autophagy. Biochem J.

[219] Trelford C.B., di Guglielmo G.M. (2022). Autophagy regulates transforming growth factor β signaling and receptor trafficking. Biochim Biophys Acta Mol Cell Res.

[220] Cai Y., Cai J., Ma Q. (2018). Chloroquine affects autophagy to achieve an anticancer effect in EC109 esophageal carcinoma cells *in vitro*. Oncol Lett.

[221] Brittain H.K., Scott R., Thomas E. (2017). The rise of the genome and personalised medicine. Clin Med Lond Engl..

